# Genome Sequencing and Comparative Genomics of the Broad Host-Range Pathogen *Rhizoctonia solani* AG8

**DOI:** 10.1371/journal.pgen.1004281

**Published:** 2014-05-08

**Authors:** James K. Hane, Jonathan P. Anderson, Angela H. Williams, Jana Sperschneider, Karam B. Singh

**Affiliations:** 1 Molecular Plant Pathology and Crop Genomics Laboratory, Centre for Environment and Life Sciences, Division of Plant Industry, Commonwealth Scientific and Industrial Research Organisation, Floreat, Western Australia, Australia; 2 The University of Western Australia Institute of Agriculture, University of Western Australia, Crawley, Western Australia, Australia; MicroTrek Incorporated, United States of America

## Abstract

*Rhizoctonia solani* is a soil-borne basidiomycete fungus with a necrotrophic lifestyle which is classified into fourteen reproductively incompatible anastomosis groups (AGs). One of these, AG8, is a devastating pathogen causing bare patch of cereals, brassicas and legumes. *R. solani* is a multinucleate heterokaryon containing significant heterozygosity within a single cell. This complexity posed significant challenges for the assembly of its genome. We present a high quality genome assembly of *R. solani* AG8 and a manually curated set of 13,964 genes supported by RNA-seq. The AG8 genome assembly used novel methods to produce a haploid representation of its heterokaryotic state. The whole-genomes of AG8, the rice pathogen AG1-IA and the potato pathogen AG3 were observed to be syntenic and co-linear. Genes and functions putatively relevant to pathogenicity were highlighted by comparing AG8 to known pathogenicity genes, orthology databases spanning 197 phytopathogenic taxa and AG1-IA. We also observed SNP-level “hypermutation” of CpG dinucleotides to TpG between AG8 nuclei, with similarities to repeat-induced point mutation (RIP). Interestingly, gene-coding regions were widely affected along with repetitive DNA, which has not been previously observed for RIP in mononuclear fungi of the Pezizomycotina. The rate of heterozygous SNP mutations within this single isolate of AG8 was observed to be higher than SNP mutation rates observed across populations of most fungal species compared. Comparative analyses were combined to predict biological processes relevant to AG8 and 308 proteins with effector-like characteristics, forming a valuable resource for further study of this pathosystem. Predicted effector-like proteins had elevated levels of non-synonymous point mutations relative to synonymous mutations (dN/dS), suggesting that they may be under diversifying selection pressures. In addition, the distant relationship to sequenced necrotrophs of the Ascomycota suggests the *R. solani* genome sequence may prove to be a useful resource in future comparative analysis of plant pathogens.

## Author Summary

The fungus *Rhizoctonia solani* is divided into several sub-species which cause disease in a range of plant species that includes most major agriculture, forestry and bioenergy species. This study focuses on sub-species AG8 which causes disease of cereals, canola and legumes, and compares its genome to other *R. solani* sub-species and a wide range of fungal and non-fungal species. *R. solani* is unusual in that it can possess more than one nucleus per cell. The multiple nuclei and sequence mutations between them made assembly of its genome challenging, and required novel techniques. We observed signs that DNA sequences originating from multiple nuclei in AG8 exhibit a high frequency of single nucleotide polymorphisms (SNPs) and more SNP diversity than most fungal populations. These SNP mutations also have similarities to repeat-induced point mutations (RIP). Moreover in AG8, RIP-like SNPs are not restricted to intergenic regions but are also widely observed in gene-coding regions. This is novel as RIP has previously only been reported in repetitive DNA of distantly-related fungi that have only a single nucleus per cell. We generated a list of 308 genes with similar properties to known plant-disease proteins, in which we found higher rates of non-synonymous mutations than normal.

## Introduction

*Rhizoctonia solani* (formerly, teleomorph: *Thanetophorus cucumeris*) is a globally-distributed, soil-borne fungal phytopathogen employing a necrotrophic lifestyle. Collectively, the host-range of the *R. solani* species spans numerous plant species vital to the agriculture, forestry and bioenergy industries, including but not limited to: wheat, rice, barley, canola, soybean, corn, potato and sugar beet [1]. Chemical control methods may not be feasible nor economical for the control of many soil-borne pathogens [2]. Hence, agronomic controls such as crop-rotation are heavily relied upon to fight this disease, though the polyphagous habit of some isolates can include commonly rotated crop species. For example, cereal and legume rotations are susceptible to AG8 [1], [3]; and corn, canola and soybean rotations are susceptible to AG1 and AG2 [4]–[5]. Susceptible crop species possess at best, low to moderate levels of genetic resistance which are of limited use to conventional breeding strategies [6]–[8]. The impact of *R. solani* has been observed to increase in incidence and severity with increased adoption of conservation (no-till) farming techniques [2].The combinations of these factors places *R. solani* as a significant threat to global food security and other agro-forestry industries.

The *R. solani* species complex is comprised of fourteen anastomosis groups (AGs), most of which are reproductively incompatible with each other and are numbered AG-1 through AG-13. The ‘bridging isolate’ AG-BI is the exception, being compatible with multiple AGs [1], [9]. Despite an apparently low level of phylogenetic divergence between AGs [10] they exhibit diverse phenotypic variation, particularly with respect to the host-ranges of phytopathogenic AGs (Supporting Table S1A). Less frequently, certain AGs have been observed to have a predominantly saprophytic or mycorrhizal life-cycle.

Our study presents a comprehensive genome assembly and functional analysis of *R. solani* AG8, causative agent of bare patch of wheat, barley and legume species [3], [11]–[12]. Of the AGs that infect wheat, AG8 is the most damaging. In Australia, the impact of *R. solani* on wheat and barley production is estimated upwards of $77 million per annum and bare patch also remains a major problem for the production of wheat and other crops in the US [13]. The host-range of the sequenced isolate WAC10335 (zymogram group ZG1-1 [14]) also extends to legume species of agricultural and scientific importance: *Lupinus* spp. (lupin) [15] and *Medicago truncatula* (barrel medic) [16], but not to the non-legume *Arabidopsis* [17]. As a basidiomycete, the plant pathogens most closely related to *R. solani* with genome sequences available are the biotrophic smuts [18]–[20], rusts [21]–[22] and the tree-pathogenic *Moniliophthora* spp. [23], which possess vastly different lifestyles. Thus, the information gained from *R. solani* is expected to be of importance in filling gaps in our knowledge of plant pathogen biology, which apart from rusts and smuts, is skewed towards the ascomycetes.

Significant genomic resources for other AGs of *R. solani* have also recently become publicly available, formerly being limited to EST libraries of AG1-IA [24] and AG4 [25]. The recent generation of whole genome sequences of *R. solani* AGs presents new opportunities for comparative genomics between *R. solani* anastomosis groups. The most comprehensive whole-genome study to date has been that of the rice pathogen AG1-IA [26] [GenBank: AFRT00000000]. The genome assembly of the closely related AG1-IB was published recently [27] [GenBank: CAOJ00000000], however full scaffold sequences were not in the public domain at the time of writing and thus AG1-IB data has not been used for synteny comparisons in this study. The mitochondrial genome sequence of the potato pathogen AG3 strain Rhs1AP and its comparison to that of AG1-IB has been published recently [28]. A draft nuclear genome for AG3 is also available (http://www.rsolani.org with kind permission from Cubeta *et al.*), however a nuclear gene dataset and genome survey have not yet been published [29].

*R. solani* AG8 exists as a multi-nuclear heterokaryon in which individual *R. solani* cells may carry multiple nuclei and copy number can vary between cells. An average of 8 nuclei per cell has previously been observed in AG8, but numbers commonly ranged from 6 to 15 [1]. While reduction of nuclear complexity via protoplast isolation has been reported for *R. solani* [30]–[32], we chose to assemble a representative haploid assembly of all AG8 nuclei in an agriculturally-relevant isolate and investigate mechanisms and type of sequence variations between nuclei in this largely asexual isolate. We report evidence of SNP-level diversity between heterokaryotic nuclei of a complex fungal genome, which has not previously featured extensively in genome studies of fungal phytopathogens. The heterozygosity between nuclei of AG8 compounded the complexity of its *de novo* genome assembly [available from GenBank: AVOZ00000000] and we also describe novel bioinformatic approaches used to overcome these challenges. This study also compares whole-genome synteny between *R. solani* anastomosis groups (AG8, AG1-1A and AG3) and uses comparative genomics techniques to highlight genes and functions unique to AG8 and AG1-1A. Predicted properties of AG8 proteins have been leveraged to generate a list of 308 ‘effector-like’ genes that may be related to plant-pathogenicity. These collective resources will be important for further experimentation in this pathosystem.

## Results & Discussion

### The haploid consensus genome assembly of *R. solani* AG8

**Figure 1:**
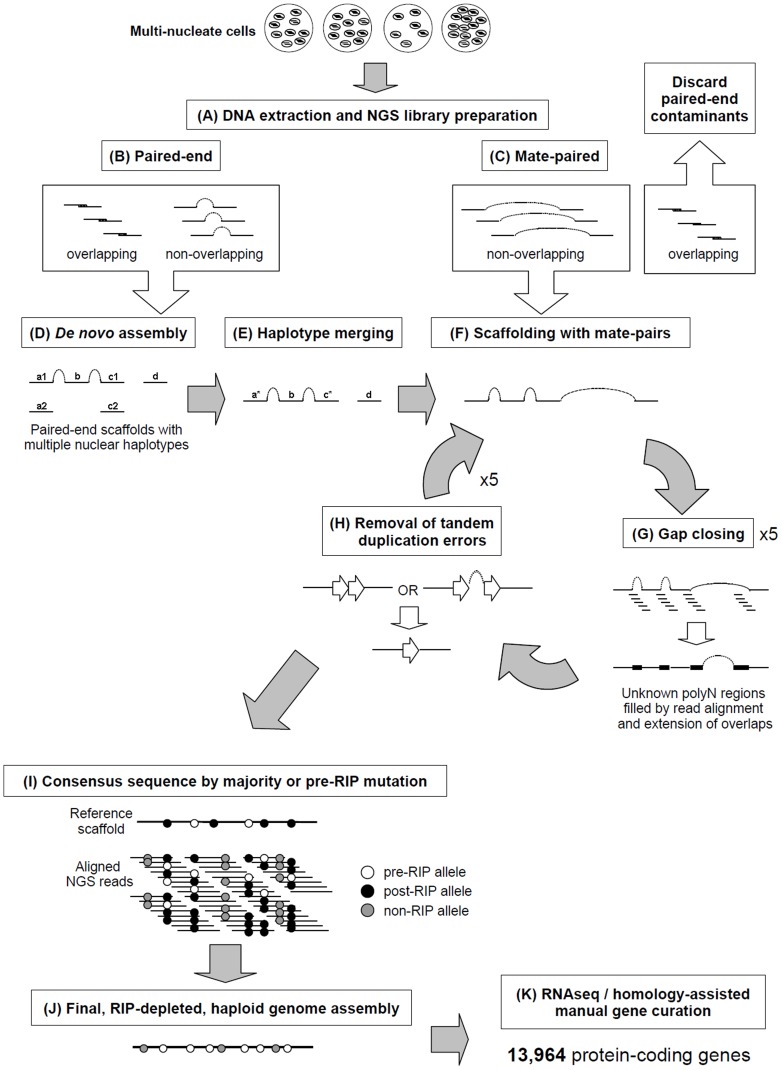
A novel pipeline was employed to assemble the multinucleate, heterozygous genome of *Rhizoctonia solani* AG8. A) Genomic DNA from multinucleate cells with variable nucleic copy numbers was prepared for next-generation sequencing (NGS) Illumina paired-end (B) and mate-paired (C) short-read libraries. The 3′ ends of read pairs from both (B) and (C) were tested for overlapping sequence, indicating short DNA fragment sizes. Overlapping pairs in mate-paired libraries (C) were discarded as these indicated paired-end contaminants which would lead to assembly errors. D) *De novo* assembly was performed combining the non-overlapping and overlapping paired-end read pairs that were merged into longer single-end reads. E) Redundant haplotypes where equivalent regions of the genome from multiple nuclei were present more than once in the assembly were merged into a single haplotype sequence. F) Non-overlapping mate-paired reads were used to build assembled sequences into larger scaffold sequences. Stretches of unknown bases (polyN) in the assembly were filled where possible (G) by alignment of genomic NGS reads to the assembly and regions predicted to contain tandem-duplication errors were corrected (H). Processes F, G and H were repeated for several rounds to ensure complete assembly. I) Minor assembly errors and the presence of RIP mutation between nuclei were corrected by substitution of the most dominant or pre-RIP allele. The final RIP-depleted, haploid consensus genome assembly (J) was manually annotated using a combination of RNA-seq and protein homology supporting evidence, producing a final dataset of 13,964 protein-coding genes (K).

**Figure 2:**
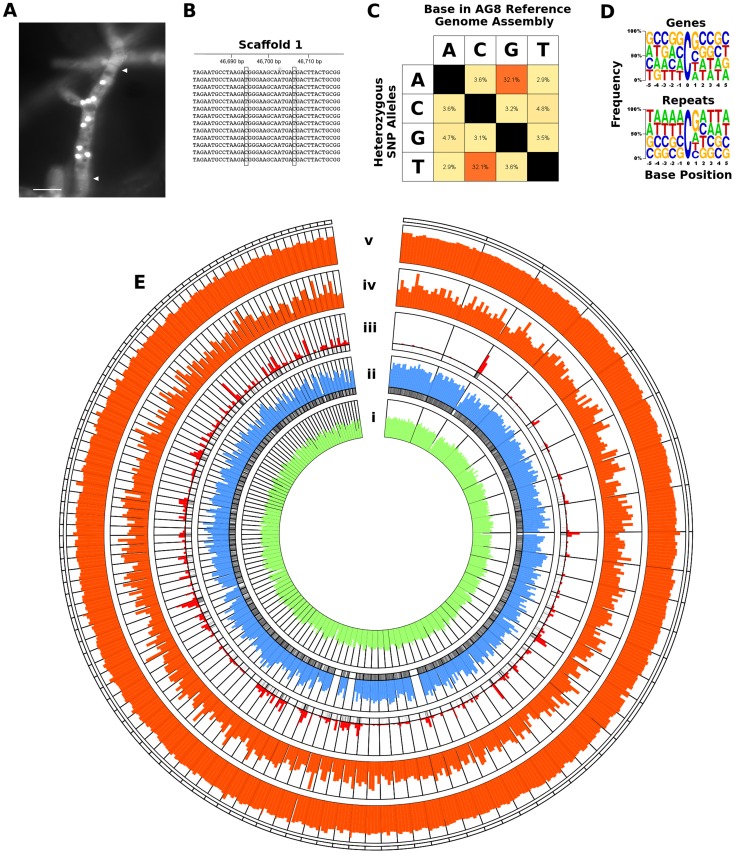
RIP-like mutation was observed across repetitive and gene-encoding regions of the *R. solani* AG8 assembly. A) Fluorescence micrograph of *Rhizoctonia solani* AG8 hyphae (stained with SYBR green) displaying multiple nuclei within a single cell. Nuclei appear as brightly fluorescent structures. Hyphal septa are indicated with arrows and the scale bar is equivalent to 20 µm. Prior to genome analysis, sequence variation between nuclei was unknown. B) Close-up view of the genomic region corresponding to actin gene *RSAG8_00181*, with short genomic sequence reads used in its assembly. This is representative of most genomic regions, in which constituent short reads exhibit two dominant haplotypes differentiated by low frequency SNP mutation. C) Percentage frequency matrix of SNP mutation type at heterozygous sites in the AG8 assembly. The majority were transition mutations between cytosine and thymine (reverse complement adenine and guanine). D) Frequency logos of the base composition of the sequences flanking heterozygous C↔T transitions in gene and repeat sequences, exhibiting a moderate bias for a 3′ guanine (i.e. CpG) in both. E) Distribution of genes, repeats and cytosine hypermutations across AG8 nuclear scaffolds of at least 100 kbp in length (scaffolds 1-76 and 78-117). All plot data in concentric rings are calculated within sequential 100 kbp windows, in order from the centre outwards: (i) G:C content (green, from 40 to 60%); (ii) percentage of 100 kbp window region covered by protein-coding genes (blue, from 0 to 100%); (iii) percent coverage of repetitive sequences (red, from 0 to 100%). The presence (black) or absence (white) of gene or repeat regions are also indicated directly below rings (ii) and (iii) respectively; (iv) frequency of heterozygous C↔T (and A↔G) polymorphisms (orange, 0 to 1000); (v) ratio of heterozygous C↔T (and A↔G) sites relative to all SNPs (orange, 0 to 100%).

The heterokaryotic nature of the *R. solani* genome posed considerable challenges for genome assembly. To overcome these challenges we developed a novel genome assembly pipeline (Figure 1). The assembly process, including software and parameters, is described in the Materials and Methods section with additional information in Supporting Text S1. Preliminary *de novo* assemblies exhibited high levels of sequence redundancy and heterozygosity across gene-encoding regions. We confirmed that multiple nuclei were present in variable numbers within cells of the sequenced isolate (Figure 2A). In order to reduce sequence redundancies caused by the assembly of heterozygous homeologs, the process used to assemble the AG8 genome included a step to merge haplotype contigs prior to scaffolding. This step was followed by generation of a haploid ‘majority consensus’ sequence from alignments of genomic sequence reads to merged scaffolds. However prior to this study, the extent of sequence variation between homeologous chromosomes originating from different nuclei was unknown. Alignment of genomic deep-sequencing reads to the genome assembly indicated an abundance of heterozygous SNP mutations throughout the AG8 assembly (Figure 2B). As many as 74% of heterozygous SNP alleles were transition mutations between cytosine and thymine (or their complementary bases guanine and adenine) (Figure 2C, Supporting Table S2A). Nucleotides flanking these C→T ‘hypermutations’ exhibited a moderate bias of approximately 40% for a G at the 3′ base (i.e. CpG→TpG) (Supporting Table S2B). These cytosine and CpG hypermutations were widespread across the AG8 genome and occurred within protein-coding genes and repetitive DNA regions at similar levels (Figure 2D), with only a slight reduction in CpG frequency in genes relative to repeats. One of the consequences of C→T mutation is the introduction of stop codons into protein-coding open-reading frames (ORFs) [33]. We reason that it is possible for ORFs to be inactivated by nonsense mutations in the majority of nuclei, yet still produce functionally active, full length proteins from a low number of non-mutated nuclei in *R. solani* AG8. Thus the assembly process also included a step which reverted heterozygous mutations between C and T to cytosine, regardless of allele frequencies. The final *R. solani* AG8 draft assembly comprises 861 scaffolds, has a total length of 39.8 Mbp which is consistent with previous haploid cytogenetic estimates of 37 to 46 Mbp [34], an N50 of 65 and an N50 length of 160.5 kbp (Table 1).

**Table 1:** Assembly statistics of four draft genome assemblies of *R. solani*.

	AG8	AG1-IA	AG1-IB*	AG3
Host range	Wheat, barley, canola, legumes	Rice, canola, maize	Rice, soybean, brassicas	Potato, sugar beet, tomato, cotton
Sequencing method	Illumina: 75 bp paired-end, 200 bp insert size,	Illumina: 100 bp paired-end, 100 bp insert-size, 75 bp paired-end, 347 bp insert size	2× GS-FLX whole-genome shotgun libraries	Sanger ABI 3730 XL: 4 and 10 kbp plasmid, 40 kbp fosmid
	100 bp paired-end, 300 bp insert size	44 bp mate-paired, 2.2 kbp, 5.3 kbp and 5.6 kbp insert size	1× GS FLX 3 kbp long-tag paired end library	1× GS FLX whole-genome shotgun library
	100 bp mate-paired, 2 kbp, 5 kbp and 10 kbp insert size			
Assembly method	SOAPdenovo 1.05SSPACE 2.1GapCloser 1.2	SOAPdenovo 1.05	GS De novo Assembler 2.6	Unknown/In progress
Isolate	WAC10335	N/A	7-3-14 (AJ868459)	Rhs 1AP
Total assembly length	39.8 Mbp	36.9 Mbp	47.65 Mb	51.0 Mbp
Average scaffold length	46,260 bp	13,949 bp	N/A	5,377 bp
Maximum scaffold length	1,192,818 bp	4,751,343 bp	1,082,565 bp	384,139 bp
Minimum scaffold length	1,003 bp	500 bp	N/A	360 bp
N50	65	19	N/A	486
N50 length	160,522 bp	474,500 bp	N/A	21,841 bp
Total number of scaffolds	861	2,648	1,600	9,484
Protein-coding genes	13,964 (manually curated)	10,489	12,268	N/A*
Predicted tRNA genes	94	102	167	N/A
Predicted ncRNA genes	155	N/A	N/A	N/A

*scaffold sequences not available for further comparison at time of writing.

**Figure 3:**
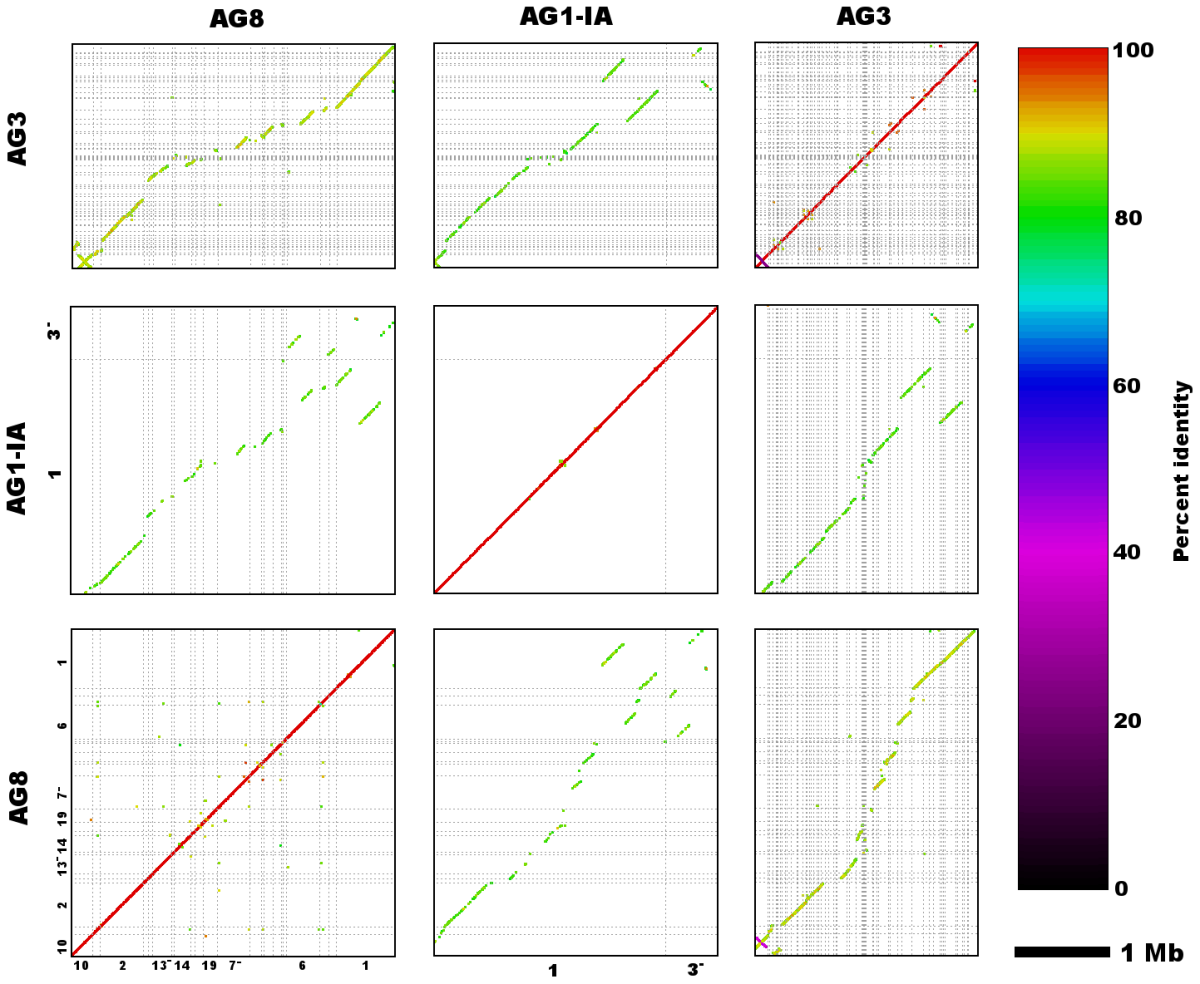
Genome assembly sequence comparisons between *R. solani* AG8 and isolates from alternate anastomosis groups. Dot-plots depict nucleotide sequence matches detected via MUMmer (nucmer) between the two largest scaffolds (both Scaffold_1) of *R. solani* AG8 and AG1-IA, as well as other homologous scaffolds from AG8, AG1-IA and AG3. Sequence alignments exhibit a predominantly co-linear, macrosyntenic configuration, however a small number of structural rearrangements can be observed between the larger scaffolds of AG8 and AG1-IA. Due to partial assembly of these genomes and thus short length of many scaffold sequences depicted here, only longer scaffolds have been labelled with their numbers along the x- and y-axes, however full details of alignments can be found in Supporting Table S3.

The AG8 genome assembly statistics compared favorably with those of other *R. solani* isolates AG1-1A, AG1-1B and AG3 as shown in Table 1. Sequence comparisons between the whole genome assemblies of *R. solani* AG8, AG1-1A and AG3 exhibited widespread co-linearity or macrosynteny [35] (Figure 3, Supporting Table S3). No conclusive evidence for dispensable chromosomes, as reported for *F. oxysporum* [36], was observed.

A single scaffold (Scaffold_77) of ∼140 kbp in length was predicted to represent the mitochondrial genome. The ends of the mitochondrial scaffold sequence were confirmed to be physically joined in a circular configuration by PCR (Supporting Text S2). The mitochondrial scaffold contained the expected set of fungal mitochondrial genes (*atp6*, *cytb*, *cox1-3*, *nad1-5* & *nad4L*, *rps5*, *rns* & *rnl*) and was abundant with LAGLIDADG and GIY-YIG intronic endonucleases. This is consistent with recent reports for the mitochondrial genomes of AG3 and AG1-IB, which are of similarly large sizes (235.8 kbp and 162.8 kbp respectively) and possess high abundances of endonucleases [28].

Within the nuclear genome, repetitive DNA sequences (Supporting Table S4A) represented just over 10% of its total length. Gypsy retrotransposons were the most abundant repeat type and represented 4% of the nuclear genome. Protein-coding gene-based tri-nucleotide simple sequence repeats, WD40-like and tetratrichopeptide repeats, represented approximately 1%. Comparing the repetitive content of AG8 with available repeat data for AG1-1A, we observed more repetitive DNA in the assembly of AG8 (10.03% of the assembly) compared to that of AG1-1A (5.27%) [26]. It should be noted that critical differences in assembly, *de novo* repeat prediction and repeat classification methods may limit the comparability of these two datasets, however the proportions of the most dominant repetitive elements was strikingly similar. The most dominant transposable elements in both AG8 and AG1-1A were LTR retrotransposons: the most common being the Gypsy/Dirs1 family at 3.98% and 3.43% respectively; followed by the Ty1/Copia family at 0.14% and 0.60% respectively. This pattern of Gypsy being more numerous than Copia retroelements, appears to be typical of most fungal genomes [37]. Non-coding RNA (ncRNA) genes were predicted *in silico* (Supporting Table S5A), which overall made up less than 0.007% (26.5 kbp) of the total genome length.

### Manual curation of 13,964 *R. solani* AG8 protein-coding genes with RNA-seq support, enables prediction of proteome and secretome properties

To enable discovery and accurate annotation of protein-coding genes present in the AG8 assembly, particularly those expressed in the presence of plant tissues, three high-coverage Illumina RNA-seq libraries were aligned to the genome to delineate gene exon boundaries. To obtain transcript data for as many genes as possible, the libraries included one library of AG8 undergoing vegetative growth in culture and two “infection-mimicking” libraries. These libraries were derived from AG8 grown on water agar containing wheat (*Triticum aestivum*) or *Medicago truncatula* seedlings separated by a permeable nitrocellulose membrane. This enabled collection of fungal tissue whilst reducing plant tissue contamination to negligible amounts. Alignment of RNA-seq data and proteins from related fungal species and pathogenicity gene databases were combined with *in silico* gene predictions to automatically predict gene structure annotations, which were then manually curated.

**Figure 4:**
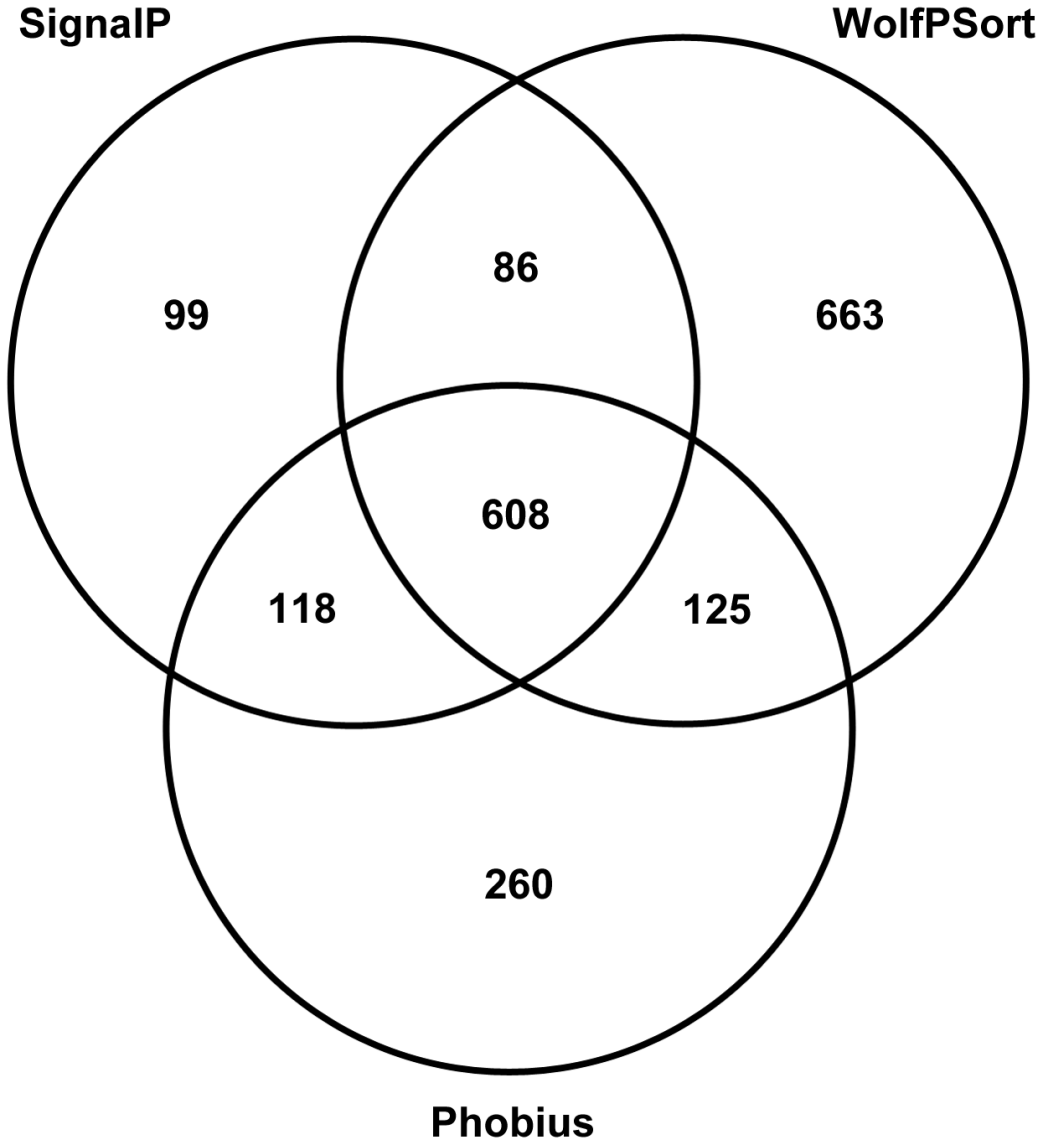
Summary of secreted proteins predicted by 3 different methods: SignalP, WolfPsort and Phobius.

The density of gene-coding regions was relatively even throughout the assembled genomic scaffolds (Figure 2E_ii_), with reduced density at some scaffold termini with high levels of repeats (Figure 2E_iii_). A total of 13,964 protein-coding AG8 genes that can serve as a reference for *R. solani* comparative genomics were predicted after RNA-seq-assisted manual gene annotation. Of these, 8,449 proteins had a BLASTP match to the NCBI NR protein database (Supporting Figure S1, Supporting Table S6). The taxonomic distribution of lowest-common ancestor taxa for these BLASTP matches indicated wide conservation of 83% (7016/8449) of *R. solani* AG8 with fungal proteins, 52.5% (4436/8449) specifically conserved within the Basidiomycota (Supporting Figure S1) and 17.9% conserved within the class Agaricomycetes. The extracellular secreted component of these proteins was predicted using a combination of SignalP [38], WolfPsort [39] and Phobius [40] (Figure 4). A total of 1,959 proteins (14.0% of all proteins) were predicted to be secreted by one or more methods and 608 (4.4%) were predicted to be secreted by all three methods. For comparative purposes, SignalP predictions were applied to *R. solani* AG8 and across 86 fungal species (Supporting Table S7). There were 911 secreted proteins predicted by SignalP for AG8, which was similar to the numbers predicted for closely-related plant-pathogenic species of the class Agaricomycetes. The secretome counts across biotrophic Basidiomycetes of other classes were relatively variable, e.g. *Puccinia striformis* (1,264), *P. graminis* f. sp. *tritici* (2,012) and *Ustilago maydis* (595). However AG8 was within a similar range to the average predicted secretome count across all fungi (1,052), which was predominantly comprised of necrotrophs.

To surmise the biological processes important to *R. solani* AG8 in the infection process, we predicted the functions of its 13,964 genes by comparison to the CAZy (Carbohydrate-Active enZyme) and Pfam (Protein family) databases. In total, we assigned CAZy annotations to 1,137 genes (Supporting Table S8B,C) and Pfam annotations to 6,099 genes (44.5%) (Supporting Table S9A).

**Figure 5:**
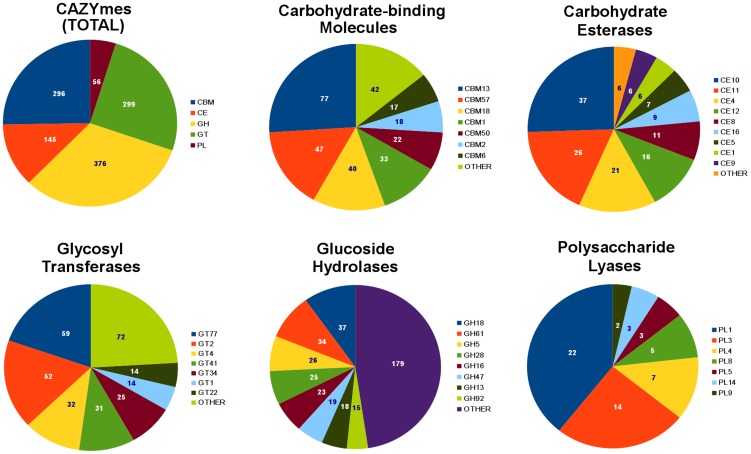
Summary of *R. solani* AG8 genes assigned with CAZyme functional annotations. An overall summary is presented for the 5 CAZyme categories: carbohydrate-binding molecules (CBMs), carbohydrate esterases (CEs), glycosyl transferases (GTs), glucoside hydrolases (GHs) and polysaccharide lyases (PLs). Individual summaries are also presented for each category, showing their most abundant CAZyme classes.

Analysis of CAZymes present in the *R. solani* AG8 genome (Figure 5) revealed a dual bias for the degradation of the structures of plant cells and modification of the fungal cell wall for growth or protection from host-defences (Supporting Table S8C). The most abundant CAZy families are described here. The most prevalent glycoside hydrolase (GH) CAZyme class (GH18) represented chitinases, followed in frequency by classes representing cellulases (GH5), polygalacturonases (GH28) and beta-glucanases (GH16), which degrade major components of plant cell walls. The most abundant glycosyltransferase (GT) classes were strongly geared towards cellulose (GT2, GT41), hemicellulose (GT77, GT4, GT34) and chitin (GT2) degradation. The most common carbohydrate esterase (CE) class contained choline esterases (CE10). Polysaccharide lyase (PL) CAZymes were strongly biased towards pectin-degradation, with the two most dominant classes (PL1 and PL3) both representing pectate lyases. The three most abundant carbohydrate-binding (CBM) class CAZymes were lectin-like proteins. Two of these (CBM13 and CBM57) are predicted to bind cellulose and hemicelluloses and include ricinB-like lectins. The third (CBM18) contains sialic-acid-binding lectins, which may play a role in protection from plant host-defenses by ‘shielding’ sugars protruding from the fungal cell wall [41]. The fourth most frequent CBM class (CBM1) binds chitin and cellulose and appears to be conserved exclusively within fungal species.

Pfam domains in *R. solani* AG8 were compared to Pfam annotations assigned to a panel of 50 pathogenic and non-pathogenic fungal species (obtained from the JGI Integrated Microbial Genomes database) (Supporting Figure S2, Supporting Table S9A) [42]. *R. solani* AG8 exhibited high abundance of tyrosine protein kinase signalling, membrane transport, protein-protein binding, reduction-oxidation, DNA methylation and a bias among cell-wall degrading enzymes towards pectin and peptidase degradation. Pfam domains with protein-protein binding functions were dominated by various classes of tetratrichopeptide repeats, but also included other domains involved in protein binding interactions: (WD40-like) PD40 beta-propeller [Pfam: PF07676]; Ankyrin [Pfam: PF13606] and leucine-rich repeats [Pfam: PF00560]. The most abundant peptidase domain was the CHAT (Caspsase HetF-Associated with TPRs) domain [Pfam: PF12770] which may be involved in programmed cell death. In summary, *R. solani* AG8 possesses a number of gene families whose members have a broad range of potential biological roles, for example those encoding caspases or protein-binding functions. Further study would be required to determine their relevance to plant pathogenicity or other lifestyle characteristics. These findings do however indicate that *R. solani* AG8 possesses a large number of carbohydrate-binding lectins of unknown function as well as a battery of CAZymes suitable for consumption of carbohydrates commonly found in cereal hosts, but also is geared towards the degradation of pectin.

### Comparison of functional annotations between AG8 and AG1-IA suggest molecular functions of importance in host-specific plant-pathogen interactions

Publicly-available protein data for AG1-IA [26] was also used to generate functional annotations for AG1-IA. Statistical comparisons between functions predicted in AG8 and AG1-IA were performed using Fisher's exact test (p≤0.05) (Supporting Table S10A). *R. solani* AG8 and AG1-IA primarily infect two different hosts - wheat and rice respectively. Differences between them in their relative abundances of functionally-annotated genes may reveal important differences in their infection strategies. Overall, fewer Pfam domains were found to be significantly higher in AG1-IA than in AG8. In AG1-IA (Supporting Table S10B), the Pfams that were significantly more abundant and may be related to pathogenicity included several types of transmembrane transporter domain and formin-like proteins that may be involved in cytokinesis. Many more functions were found to be increased in AG8 relative to AG1-IA (Supporting Table S10C), however most of these were of too broad or unknown function to infer their biological roles. Nevertheless, several functions stood out as potentially important for plant pathogenicity in AG8, including CAZymes, peptidases, membrane transporters, transcription factors and toxin-like proteins. Peptidases abundant in AG8 included the CHAT and C14 domain caspases as well as fungalysin-like peptidases. The CAZyme functions that were significantly more numerous in AG8 were predominantly glycosyl-hydrolases (polygalacturonases, β-galactosidases), pectate lyases and carbohydrate binding proteins (ricin-like and jacalin lectins and fungal-specific CBM1 proteins). Fungal pathogens of dicots generally possess higher numbers of pectin-degrading enzymes than monocot pathogens [43]. Though an important pathogen of monocot cereals, most notably wheat, the sequenced isolate of *R. solani* AG8 was isolated from the dicot lupin and is also an important pathogen of other leguminous dicots. The abundance of pectate lyases in AG8 relative to AG1-IA is likely to reflect the broad host range of the sequenced AG8 isolate.

Interestingly, AG8 had more members of two Pfams similar to ricinB lectins [44] and delta endotoxins [45], highly toxic proteins commonly associated with defence against insect predators which have been prioritised for further study. In contrast to AG1-IA which had none, AG8 possessed 3 delta-endotoxin-like proteins (RSAG8_06697, RSAG8_07821 and RSAG8_07820) with the Pfam domain Bac_thur_toxin [Pfam: PF01338]. This domain was originally defined based on the insecticidal delta endotoxins of *Bacillus thuringiensis*. Pfam matches and orthology analysis suggested the presence of orthologous delta endotoxin-like proteins in other phytopathogenic species including *Fusarium graminearum* (Fusarium head blight of wheat and barley) and the bacteria *Dickeya dadantii* (syn. *Erwinia chrysanthemi*, soft-rot, wilt and blight on a range of plant hosts and septicaemia of pea aphid) [46] (Supporting Table S9A, Supporting Table S11). Whether these ricinB and delta-endotoxin homologs confer an advantage against competitors or predators or may instead be toxic to the plant host remains to be determined.

### Prediction of 308 *R. solani* AG8 plant-pathogenicity gene candidates

Effector proteins have been observed to be secreted by several microbial pathogens [47] and cause disease on their respective hosts. A set of characteristics common to plant pathogenicity effectors from fungi that would allow reliable bioinformatic predictions has not yet been accurately defined. However experimentally validated effectors tend to be low molecular weight, secreted, cysteine-rich proteins which may contain certain conserved amino-acid motifs near the N-terminus [47]–[48] (Supporting Table S12). Effector-like proteins were predicted in AG8, requiring: complete annotation from translation start to stop with <3 consecutive unknown (‘X’) amino acids; predicted molecular weight ≤30 kDa; predicted as secreted with 0–1 predicted transmembrane domains; and with ≥4 cysteine residues. A total of 308 AG8 proteins matched all of these criteria. These candidates were searched for known motifs previously associated with plant pathogenicity, however the occurrence of these motif matches was not significant relative to the complete protein dataset. As an example the RxLR-like motif (Kale et al., 2011), though found in 73% of the predicted effector candidates, was also found in 77% of the whole *R. solani* AG8 proteome (Supporting Table S13) indicating this permissive motif may not be useful for effector candidate prediction in *R. solani* AG8. We were also unable to identify any novel N-terminal-associated motifs that were highly conserved among these 308 proteins (Supporting Text S3). However, we observed the ratio of non-synonymous to synonymous mutations (dN/dS) within these 308 candidate genes to be 0.97 compared to 0.77 across all genes. Our understanding of the roles of these 308 effector candidates will benefit from the addition of further experimental data, resulting in a more succinct list of candidates with a potential direct role in disease on one or more of the many plant hosts of *R. solani* AG8. Unfortunately, no method for the stable transformation of *R. solani* AG8 is presently available and thus functional testing of candidate pathogenicity genes will be challenging.

To gain further support for an association with pathogenicity, approximately 10% (29) of the 308 predicted ‘effector-like’ genes were randomly selected and their mRNA expression relative to a set of 7 constitutively expressed genes was compared between *R. solani* AG8 sampled at 7 days post-infection of wheat and 7 day-old AG8 mycelia grown on media. Of these 29 genes, 25 (85%) had a positive fold-change and 17 had a significantly higher relative expression *in-planta* (Student's t-test; p≤0.05, log2 fold change ≥1) (Supporting Table S14B). This dataset highlights several plant-pathogenicity candidates, but other genes also important for pathogenicity may not be changing in abundance during infection relative to *in-vitro* growth.

### Widespread CpG-biased hypermutations may be similar to repeat-induced point mutations (RIP) observed in mononuclear species

Repeat-induced point mutations (RIP) are fungal-specific SNP mutations previously reported to be restricted to the filamentous Ascomycota (Pezizomycotina) [49]. RIP in the Pezizomycotina involves transition mutations converting cytosine to thymine, with a moderate bias for CpA dinucleotides [49]. Other features of RIP include targeted mutation of repetitive DNA, with single-copy DNA regions being largely unaffected. An important exception to this is where RIP mutations ‘leak’ into single-copy DNA regions from neighbouring repetitive DNA which occurs more frequently closer to repeats [50].

The small number of studies looking for RIP-like mutations in the Basidiomycota do not exhibit the characteristic CpA mutation bias observed in the Pezizomycotina [49], however two studies have reported a CpG dinucleotide bias between repetitive DNA sequences within the Basidiomycota and a TpCpG trinucleotide bias specific to the subphylum Pucciniomycotina [51]–[52]. As an Agaricomycete, we expect *R. solani* to exhibit a bias towards CpG but not TpCpG. However, it should also be noted that hypermutations of CpG may also be caused by widely conserved processes involving the methylation of cytosine to 5-methylcytosine (5mC) and subsequent deamination which converts 5mC to thymine [53]. Importantly, conversion of cytosine to thyimine via methylation and deamination does not actively target repetitive DNA or ‘leak’ in the same manner as RIP.

**Figure 6:**
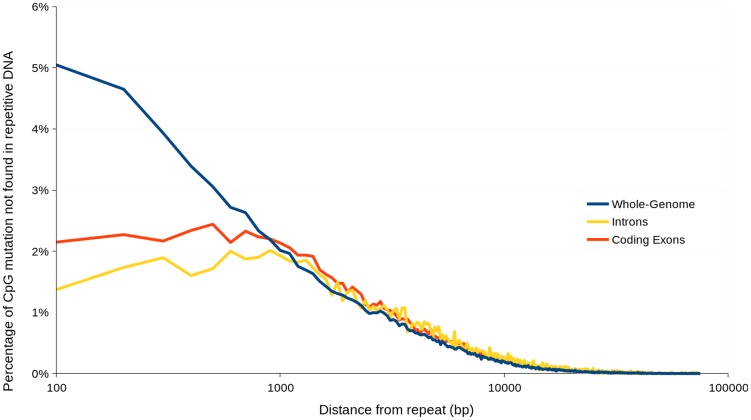
Percentage of heterozygous CpG↔TpG mutations not occurring in repetitive DNA versus distance from nearest repetitive DNA region. Percentage values were calculated based on mutations contained within incremental distance ranges of 100

Analysis of nucleotides immediately flanking heterozygous C↔T SNP sites in AG8 exhibited a CpG dinucleotide bias consistent with previous observations of ‘RIP-like’ cytosine hypermutations in the Basidiomycota [52] (Figure 2D). The distribution of these RIP-like mutations in AG8 was observed to occur across repetitive and gene-encoding regions alike at a relatively constant ratio versus non-RIP-like mutations, where heterozygous C↔T alleles comprised ∼70–80% of all SNP mutations (Figure 2E_iv-v_) and in turn CpG dinucleotides comprised ∼40–50% of heterozygous C↔T alleles. In mononuclear fungal genomes, RIP has previously only been observed to act upon repetitive DNA or to ‘leak’ into adjacent non-repetitive sequences [50]. Due to the novel genome assembly process for AG8 which involved merging of redundant haplotypes, a survey of SNP mutations in its annotated repetitive DNA would likely lead to incorrectly inflated counts of RIP-like mutations. Therefore we instead looked at the frequency of CpG↔TpG mutations versus their distance from the nearest repeat, which indicated that CpG mutations were more frequent closer to repeats (Figure 6). Furthermore, although the ratios of (C↔T/all SNPs) and (CpG↔TpG/C↔T) were relatively similar between genes and other regions of the genome, the frequency of mutations in gene regions were lower than in the genome as a whole, suggesting strong selection pressures to retain protein function. The ratio of CpG/CpH (where H = not G) was slightly lower in repeats (0.3) than in genes (0.4) (Table 2) and we speculate that this likely to be due to complete (i.e. homozygous) conversion of C→T occasionally occuring across all copies of a repeat, as they are under no selection pressure to retain their pre-RIP sequences. Thus there would be fewer sites that can be detected as heterozygous SNPs by aligning genomic reads to the genome assembly.

**Table 2:** The average distance between various types of SNP mutations, within the AG8 genome assembly, genes, predicted ‘effector-like’ genes and repetitive DNA.

Mutation type	Whole genome	All genes	Predicted effector-like genes	Repetitive DNA
All SNPs	70 bp	63 bp	55 bp	86 bp
CpN	102 bp	89 bp	81 bp	137 bp
CpA	422 bp	359 bp	337 bp	490 bp
CpC	214 bp	185 bp	165 bp	301 bp
CpG	370 bp	307 bp	265 bp	583 bp
CpT	491 bp	417 bp	386 bp	539 bp
CpH	141 bp	126 bp	116 bp	178 bp
CpG : CpH	0.4	0.4	0.4	0.3

Regardless of whether the underlying process is similar to RIP or not, CpG-biased hypermutation is likely to play an important role in the evolution of the AG8 genome. RIP has been recently proposed to have the potential to randomly introduce nonsense mutations, converting longer secreted proteins into small, secreted proteins thus making them gradually more effector-like [54]. Stop-codon frequency across the 12,771 annotated AG8 genes possessing stop codons is highest for TGA (40%) compared to TAA (31%) and TAG (29%). As TGA stop codons would be the primary nonsense product of CpG-biased hypermutation, similar evolutionary processes may also occur in AG8. Furthermore, the presence of multiple nuclei in AG8 could potentially compensate for loss of gene function due to hypermutation in one or more nuclei, allowing for a higher tolerance for the accumulation of mutations in gene-coding regions. Analysis of total SNP, and CpN dinucleotide frequencies (expressed in Table 2 as average distance in bp between mutations), showed that a SNP mutation occurred on average every 70 bp, cytosine hypermutations occurred every 89 bp and that there was a 40% bias towards CpG mutations occurring every 307 bp. Within the 308 predicted ‘effector-like’ genes, SNP mutations occurred on average every 55 bp, cytosine hypermutations every 81 bp and CpG mutations occurred every 265 bp. However, the ratios of (C↔T/all SNPs) and (CpG↔TpG/C↔T) were not significantly different between the complete set of 13,964 AG8 genes and the 308 effector-like genes. Interestingly, despite apparently similar mutation ratios, the ratio of non-synonymous to synonymous SNP mutations (dN/dS) was 0.97 in ‘effector-like’ candidates compared to 0.77 across all genes. This may suggest that the increased mutation rate conferred by CpG-biased hypermutation is advantageous for accelerating the adaptation of pathogenicity genes which, if being actively counter-acted by plant defences, are likely to be under diversifying selection.

### Comparison of SNP-level diversity within the *R. solani* AG8 whole-genome with diversity in other species

The density of heterozygous SNP mutations within AG8 was compared to SNP densities between the genome assemblies of AG8 and AG1-IA, AG1-IB and AG3 (Table 3). SNP density in AG8 was highest within intronic regions (19.6 SNPs/kbp), moderate in coding exons and genes (14.5–15.9 SNPs/kbp) and lowest in intergenic regions (11.5 SNPs/kbp). Comparisons of SNP mutations between AG8 and alternate AGs exhibited an approximately ten-fold increase in SNP density compared to the rate of heterozygous SNPs within AG8. The corresponding values within for comparisons between AG8 and AG1-IA ranged from 162.8–228.2 SNPs/kbp, AG1-IA from 141.6–200.3 SNPs/kbp and AG3 from 98.5–145.3 SNPs/kbp. We note however that in these comparisons between the genome assemblies of AG8 and other AGs, it was not possible to ascertain whether these SNPs (or homologous bases) were homozygous or heterozygous in the alternate AG. Nevertheless a higher SNP density between the AG8 genome and those of the other three AGs, relative to heterozygous AG8 SNPs, was consistent in all three comparisons.

**Table 3:** The frequency and relative densities of heterozygous SNPs within the *R. solani* AG8 genome assembly and SNPs between the genomes of AG8 and isolates from other anastomosis groups.

Anastomosis Group compared to AG8	Type	Genes	Coding exons	Introns	Intergenic regions
**AG8 (same isolate)**	regions tested (bp)	20503767	13464568	3667188	15284359
	#SNPs	326180	195631	71965	175032
	SNPs/kbp	15.9	14.5	19.6	11.5
**AG1-IA**	regions tested (bp)	2502163	5890599	1871646	389538
	#SNPs	442164	959137	427195	66890
	SNPs/kbp	176.7	162.8	228.2	171.7
**AG1-IB**	regions tested (bp)	1217710	5038424	1702580	321017
	#SNPs	191835	713605	341024	44540
	SNPs/kbp	157.5	141.6	200.3	142.7
**AG3**	regions tested (bp)	7610668	9721494	2903912	1158968
	#SNPs	866515	957888	421808	144493
	SNPs/kbp	113.9	98.5	145.3	124.7

Statistics are presented relative to genes, coding exons, introns and intergenic regions (sequences between 2 adjacent genes) in the *R. solani* AG8 genome. Comparisons between AG8 and alternate anastomosis groups were restricted to AG8 regions that aligned to alternate AG sequences across their full length.

Comparisons between individual genomes and fungal population genetics studies were also used to place the SNP diversity within *R. solani* AG8 into a wider context. Similar to AG8, the Basidiomycete stripe rust fungus *Puccinia striformis* is heterokaryotic but exhibits a lower SNP density within its genome assembly of 5.98 SNPs/kbp [21]. It may be significant that *P. striformis* is binucleate and therefore only possesses 2 nuclei per cell as opposed to the 6–15 nuclei that have been observed within cells of *R. solani* AG8 [1]. Similarly, SNP variation across a population of shiitake mushroom (*Lentinula edodes*) was reported to be 4.6 SNPs/kbp (186,0789 SNPs in 40.2 Mbp) [55]. In barley powdery mildew (*Blumeria graminis*), the SNP rate observed between pairs of isolates was lower at 1 SNP/kbp [56]. Across isolates of the multinucleate endomycorrhizal Glomeromycete *Rhizophagus irregularis* [57] and the beetle-symbiont *Leptographium longiclavatum* [58], even lower SNP densities of 0.2 SNPs/kbp (28,872 SNPs in 140.9 Mb) and 0.6 SNPs/kbp (17,266 in 28.9 Mbp) respectively, were observed. In contrast, a population study of the multinucleate human pathogens *Coccidioides immitis* and *C. posadasii* reported a rate of 23.7 SNPs/kbp relative to the *C. immitus* RS reference genome assembly (687,250 SNPs in 28.95 Mb) [59], which though slightly higher is within a similar range to *R. solani* AG8 (Table 3). In conclusion, the SNP diversity in *R. solani* AG8 appears to be higher than that observed thus far within individual isolates of binucleate rusts, between isolates of the same pathogenic species and across non-pathogen populations. Furthermore, diversity within *R. solani* AG8 is comparable to a population of another multinucleate pathogen (*C. immitus*) and much higher than that observed within a population of a multinucleate non-pathogen (*R. irregularis*). We speculate that the combination of multinuclearity and selection pressures relating to pathogenicity may be driving the accumulation of widespread heterozygous SNP diversity in *R. solani* AG8.

### Conclusions

In this study, we present a novel bioinformatics pipeline for the accurate and comprehensive assembly of a complex fungal genome, the heterozygous and multinucleate pathogen *Rhizoctonia solani* AG8 (Figure 1). The combination of genome and transcriptome sequencing allowed for data-driven gene prediction and comparative genomics with other publically available genomes of alternate anastomosis groups and other fungal species. Using a combination of novel genome assembly methods, RNA-seq, manual gene curation and comparative genomic techniques, a list of 308 ‘effector-like’ plant-pathogenicity gene candidates has been predicted. Analysis of mRNA expression for a subset of candidate pathogenicity genes during infection of wheat has highlighted several candidates for further study. Additionally, comparisons to available data for alternate AGs of *R. solani* have highlighted important differences, which may be related to differing host ranges, host tissue preference and environmental stress tolerance. The resources presented here should provide powerful tools for the identification of host-specialised mechanisms for fungal-plant interactions and pathogenicity for this important group of fungal pathogens.

CpG-biased hypermutations were observed between nuclei of AG8, within genes and repeat sequences alike and have some similarities with repeat-induced point mutation (RIP). Previous observations of RIP in haploid fungal genomes have only reported its activity upon repetitive sequences [49], [52] or non-repetitive regions within a finite distance of a repeat [50]. Although we observed hypermutation within genes, intriguingly these mutations were more numerous with increasing proximity to repeats, suggesting that repeats are mutated more frequently than genes and that a process similar to ‘RIP-leakage’ may occur. Furthermore, the molecular mechanisms of RIP have not yet been fully characterised [60] and the consequences of combining RIP-like hypermutation and multinuclearity in *R. solani* are unknown. In the basidiomycete human pathogen *Cryptococcus neoformans*, increases in ploidy and the accumulation of mutations have been implicated as mechanisms for its adaptation to immune- and drug-related selection pressures [61]. Also of note is that across isolates of the human-pathogenic and multinucleate ascomycete *Coccidiodies immitus*, higher relative frequencies of repeat-associated CpG mutation have also been observed [59] (unusual for species of the Pezizomycotina which typically exhibit a bias towards mutation of CpA [49]). We speculate that RIP-like SNP mutations accumulating in multiple nuclei may similarly be a means by which *R. solani* is also able to rapidly generate allelic diversity despite being predominantly clonally propagated [1]. Loss-of-heterozygosity and copy-number variation analyses to confirm this hypothesis would require further study using a sequencing platform which can produce longer read lengths and higher base-call accuracies than those used in this study. However, if this is the case, this mechanism may be a factor contributing to the relatively mild effectiveness of fungicide treatment against this pathogen [62] and its adaptation to a broad range of plant hosts.

## Materials and Methods

### Isolation and sequencing of *R. solani* AG8

*R. solani* AG8 isolate WAC10335 was isolated from lupin and provided by the Department of Agriculture and Food of Western Australia (DAFWA). Anastomosis group was confirmed by ribosomal ITS sequences and host-range was confirmed by inoculation assays on wheat, lupin and *Medicago truncatula* [3]. *R. solani* does not readily produce sexual or asexual spores thus single spore isolation was not possible, therefore a single rapidly growing hyphal tip was excised from a colony growing on PDA and transferred to water agar containing 250 µg/ml cefotaxime. Pathogenicity of the resulting culture was confirmed as equivalent to the original. A pure *in vitro* culture of *R. solani* was produced by incubation in PDB at 25°C with gentle shaking for 7 days. Hyphae were filtered from the culture through sterile Miracloth and rinsed with sterile water. DNA was purified by grinding hyphal tissue in liquid nitrogen and suspension in DNA extraction buffer (2% (w/v) CTAB, 1.4 M NaCl, 0.2% (v/v) β-mercaptoethanol, 20 mM EDTA and 100 mM Tris-HCl) and mixing at 60°C. Following two rounds of chloroform/isoamylalcohol extraction, the aqueous supernatant was treated with RNase I (Invitrogen) at 20 µg/ml. The DNA was purified through an additional two rounds of chloroform/isoamylalcohol extraction and precipitated by adding 0.1 volumes of 3M NaOAc (pH 5.2) and 0.6 volumes isopropanol. The resulting DNA pellet was resuspended in 10 mM Tris-HCl (pH 8.0) buffer and quantitated by Qubit (Invitrogen) and BioAnalyser prior to sequencing.

### Next-generation sequencing and pre-assembly data quality control

Two Illumina paired-end libraries of genomic DNA were sequenced, with 75 and 100 bp read lengths and median insert lengths of 250 bp and 300 bp respectively. Three Illumina genomic mate-pair libraries with insert lengths of 2 kbp, 5 kbp and 10 kbp were also obtained. Paired-end libraries were combined and trimmed for sequencing adapter/primer sequences, low-quality (<Q30), and low-complexity sequences via CutAdapt v1.1 [63] filtered for adapter sequences from the Truseq RNA and DNA sample preparation kits versions 1 and 2. Pairs with one or more reads ≤50 bp after trimming were discarded. Where possible, overlapping 3′ ends between pairs were merged into long singleton reads via FLASH v 1.2.2 [64]. FLASH was also applied to the mate-paired libraries, to remove paired-end contamination of incorrect insert length and pair orientation which would complicate genome assembly (Supporting Text S1).

For the purpose of gene annotation, Illumina paired-end libraries of 100 bp read lengths were obtained from 3 mRNA libraries derived from AG8 grown under: vegetative conditions (7 days at 25°C in PDB with gentle shaking) (non-infection) and; *Medicago* or wheat infection-mimicking conditions. Under infection-mimicking conditions, AG8 was cultured on a film of nitrocellulose overlaid on water agar containing young sterilised *Medicago truncatula* or wheat seedlings. After seven days incubation at 25°C the film and hyphae were removed, ensuring negligible plant contamination in subsequent RNA extractions with TRIzol (Sigma-Aldrich, St. Louis, MO). Two sequencing libraries were generated per mRNA library, with 200 bp and 500 bp insert sizes. Transcript libraries were trimmed for contaminant sequences via Cutadapt v1.1 as per genomic reads.

### Genome assembly

Complex genome structure caused by heterozygosity and multinuclearity prevented the use of commonly employed *de novo* assembly methods. To this end, a novel pipeline was developed for AG8 (Supporting Text S1). Paired-end libraries were assembled with SOAPdenovo v1.0.5 (k-mer length = 61) [65]. This assembly was scaffolded with SSPACE 2.0 using the parameters (end extension, min size 500 bp) [66] and subject to 5 rounds of Gapcloser2 [65] using paired-end and 3′ end merged single-end reads. Mate-paired reads were used for scaffolding but excluded from gap-closing to avoid introducing inversion errors (Supporting Text S1). Haplotype redundancy was reduced using HaploMerger v20111230 [67] (batchD: filterAli = 0; minlength = 10 bp; maxInternal = 10000000; mincoverage = 0). Tandem duplication assembly errors (common to polyploid assemblies) were corrected by a twofold approach (Supporting Text S1). The first method involved intra-scaffold re-assembly between rounds of scaffolding and gap-closing, where gaps were broken and tested for overlap via CAP3 [68]. The second method involved self-alignment via BLASTN [69], applied after scaffolding, gap-closing and N-breakage rounds had completed. Alignments occurring in tandem on the same sequence were identified, and the sequences of the second repeat plus the intermediate region were removed from the assembly if repeats were ≤500 bp apart or ≥30% polyN in intermediate region. Introduction of errors by these processes was corrected by re-alignment of raw genome reads with bowtie2 v 2.0.5 [70] followed by local-realignment at indels, variant-calling and variant-consensus generation via GATK v1.6.11 [71]. Variant Call Format (VCF v4.0) tables of SNP and indel variation between the paired-end, 3′-end merged (long single-end reads), 2 kbp mate-paired, 5 kbp mate-paired and 10 kbp mate-paired sequence libraries relative to the genome assembly sequences, were merged with VCFtools v0.1.6 [72] where variants agreed between at least 2 out of the 5 libraries. The most frequent alleles in the merged VCF were incorporated into the consensus sequence of the final assembly, with the exception of sites where cytosine (C) to thymine (T) (reverse complement: G to A) polymorphisms were observed at which the assembly was reverted to the C (or G) allele regardless of allelic frequency. The genomic distribution of SNP mutations was calculated using BEDTools v0.1.7 [73].

Genome assembly sequences of AG8, AG1-IA and AG3 were compared using MUMmer 3.0 [74] using both nucmer and promer (parameters: –maxmatch). Summary statistics were derived from coordinate outputs.

### Repetitive DNA prediction, annotation and curation

Repetitive sequences were predicted via RepeatScout v1.0.5 [75], requiring consensus sequences ≥50 bp and ≥5 copies. Full-length repeats were reconstructed from RepeatScout outputs with CAP3 (v10.15.7, -h100 -p80 -z1) [68], manually curated and mapped to the genome assembly via RepeatMasker v3.2.9 (parameters: -e crossmatch -s) [76]. Repeat types were characterised using a combination of BLASTn vs NCBI Nucleotide, BLASTx vs NCBI Protein [69], CENSOR vs REPBASE v17.11 [77] and TEClass [78]. Repeat regions were also predicted with TransposonPSI v08222010 [79] and RepeatMasker vs REPBASE v17.11 (species = “Eukaryota”) [80]. All repeat data, excluding repeats corresponding to protein-coding genes (Supporting Table S4B), were used as negative support for gene annotation.

### Transcriptome alignment and assembly

Exon splice-junctions were determined by aligning six RNA-seq libraries to the AG8 assembly via TopHat 2.0.4 (minimum intron size 20 bp, maximum intron 5000 bp, no coverage search, 2 splice mismatches, microexon search enabled, very sensitive, 20 read mismatches, 3 segment mismatches, max insertion length 12 bp, max deletion length 12 bp, report discordant and secondary alignments) [81]. Transcriptome *de novo* assembly was performed via Trinity r2012-03-17 (k-mer trimming with JellyFish 1.1.4, jaccard clipping, minimum contig length 150 bp, min k-mer coverage 5×, minimum glue 5, minimum percent read mapping 70%) [82]–[83]. Combined and individual library-specific assemblies were used for manual gene annotation.

### Gene prediction, annotation and curation

The library-specific and the combined transcriptome assemblies were used to determine exon structure using PASA r2012-06-25 (minimum percent aligned 75%, maximum intron length 5000 bp). The output of PASA was passed to the EvidenceModeller r2012-06-25 auto-annotation pipeline [84], which also incorporated the following supporting data: splice-junctions determined from RNA-seq alignment to the genome assembly via Tophat 2.0.5; *in silico* gene prediction via GeneMark-ES v3.2.3 [85]; ESTs and proteins of previously sequenced fungi and PHIbase v3.4 [86] aligned to the genome assembly with AAT r03052011 [87]; predicted repetitive DNA (see above) and; non-coding locus predictions via tRNAscan-SE v1.23 (genomic, COVE only) [88] and Infernal v1.0 [89]. Gene annotations were evaluated by EvidenceModeller, visualised in Apollo v1.11.6 [90] and manually curated. Predicted protein translations were compared to the NCBI NR Protein database by BLASTP [69] (BLAST v2.2.26, e-value≤1e^−3^, top 20 hits) and the taxonomic distribution of their corresponding lowest-common ancestor taxa was summarised with MEGAN5 (LCA parameters: minimum support 1, minimum score 40, max expected 1e^−3^, top percentage 100) [91].

### Functional annotation

Conserved protein domains in AG8 and AG1-IA were predicted with HMMER 3.0 [92] versus Pfam(A) v26.0 [88] with gathering cutoffs. Carbohydrate-active enzymes (CAZymes) were predicted with CAT v1.8 [93]. Multiple alignments of the most abundant CAZyme families were generated with MAFFT L-INS-i v7.130 [94] (Supporting Table S15). Comparison of Pfams were performed between *R. solani* AG8 and other sequenced fungi from JGI IMG v4 [42]. Orthology comparisons between AG8 predicted proteins and protein datasets from 197 fungal, oomycete, prokaryotic, insect and nematode species and included a range of pathogens with different host ranges and non-pathogens (Supporting Table S11) was performed via ProteinOrtho v4.26 (BLASTP v2.2.26, E-value = 1e-^05^, alg.-conn. = 0.1, coverage = 0.5, percent_identity = 25, adaptive_similarity = 0.95, inc_pairs = 1, inc_singles = 1, selfblast = 1, unambiguous = 0) [95]. Predicted secretome comparisons were performed using SignalP 4.1 [38] between *R. solani* AG8 and 86 other fungi (Supporting Table S7).

Candidate ‘effector-like’ pathogenicity genes were classified by: complete annotation with translation start and stop codons and ≤3 consecutive unknown ‘X’ amino acids; predicted to be secreted by at least one method; 0–1 predicted transmembrane domains (single domains can be mis-predicted within secretion signal peptides); predicted molecular weight ≤30 kDa; and ≥4 cysteine amino acids. Molecular weights and amino-acid compositions were predicted with Bio::Tools::SeqStats (BioPerl) [96]. Sub-cellular localisation, secretion status and transmembrane domains were predicted with Phobius v1.01 [40], SignalP v4.1 [38] and WolfPSort v0.2 [39]. Matches to motifs previously associated with plant pathogenicity effectors (Supporting Table S13) were searched with PREG [97] (Supporting Table S13). We also attempted to identify high frequency novel motifs within the ‘effector-like’ candidates with MEME v4.9.1 (model = ANR, minsites = 2, maxsites = 300, nmotifs = 50, minwidth = 5, maxwidth = 50) [98] (Supporting Text S3).

Heterozygous SNP mutations derived from genomic read alignment to the final genome assembly, as described above, within all genes and predicted ‘effector-like’ genes were tested for: 1) stop-codon bias; 2) gene structure location bias with SNPeff [99]; 3) non-synonymous vs synonymous SNP ratio (dN/dS) via SNPeff [99]; 4) frequency and density via BEDtools coverageBed [73].

### Validation of expression of predicted plant-pathogenicity genes during wheat infection

Gene expression of selected genes (Supporting Table S14A) was tested via quantitative polymerase chain reaction (qPCR) in wheat roots at 7 days post-infection and in 7 day old *in vitro* grown PDB culture. Wheat samples were inoculated with millet seeds pre-infect with WAC10335 and grown in pots of vermiculite for 7 days at 24°C. Wheat seeds were surface-sterilised and germinated on moist filter paper at 4°C for 4 days, then planted into pre-infected vermiculite pots and covered by a layer of fresh fine vermiculite. The pots were transferred to a growth room at 16°C and 12 hours light/day (150 µmol.m^−2^.s^−1^) for 7 days. Plants were harvested and root and above ground tissues separated. RNA was extracted from root tissue using Trizol reagent (Sigma) according to the manufacturer's instructions and cDNA produced using Superscript III (Invitrogen) following the manufacturer's instructions. Quantitative PCRs used SsoFast EvaGreen Supermix (BioRad).

A total of 29 out of 308 predicted ‘effector-like’ pathogenicity genes were selected for testing based on their assigned functional annotations. Seven control genes were also selected based on stable expression, averaging ≥70 FPKM and ≤0.1× fold change between libraries, across the three RNA-seq libraries discussed in this study and/or for putative functions suggesting stable expression patterns (e.g. actin and tubulin). Primer pairs were designed from coding-exon sequences (CDS) using primer3 [100] (opt. amplicon 200 bp, primer 18–25 bp, opt. Tm 60°C, max. ΔTm 1°C, min. GC clamp 2 bp, max. homopolymer 3 bp). *In silico* PCR screening via e-PCR [101] required ≤1 amplicon (10 bp to 10 kbp) versus genome assembly and CDS sequences. Quantitative PCR was performed with 2 technical replicates and 3 biological replicates. Log2 fold-changes between *in-vitro* and infection samples were calculated by the ΔΔCT method in accordance with Anderson *et al.* [102], relative to the mean of 7 controls. A two-tailed Student's T-test was applied to relative abundances between *in planta* and *in vitro* samples (equal variance, p-value≤0.05).

## Supporting Information

Figure S1 Summary of “lowest-common-ancestor” taxa assigned to 8,449 *R. solani* AG8 proteins by BLASTP to NCBI Protein. Higher level taxa contain protein counts both for widely-conserved *R. solani* AG8 proteins for which that taxon has been assigned as its lowest-common-ancestor, as well as cumulative counts for all lower-level taxa contained within.
(TIF)

Figure S2 Pfams abundant in *R. solani* AG8 compared to species from JGI Integrated Microbial Genomes.
(TIF)

Table S1 Summary of known *R. solani* anastomosis group host-ranges (A) and supporting publications (B).
(XLSX)

Table S2 Heterozygous SNP (A) and dinucleotide (B) mutations in the *R. solani* AG8 genome assembly.
(XLS)

Table S3 Whole-genome alignments between assemblies of *R. solani* AG8, AG1-IA and AG3 depicted in Figure 3.
(XLSX)

Table S4 Repetitive DNA content of *R. solani* AG8. (A) Summary tables of repeat proportions within the *R. solani* AG8 genome assembly. (B) Curated classifications of repeat types. (C) Genomic coordinates of repetitive DNA regions mapped by RepeatMasker. (D) Repeat predictions made via TransposonPSI. (E) Consensus sequences of *de novo* predicted AG8 repeat families in FASTA format.
(XLS)

Table S5 Summary of non-coding RNA regions predicted within the *R. solani* AG8 genome assembly. (A) ncRNA predictions made via Infernal and their genomic coordinates. (B) Summary of Infernal predictions. (C) tRNA predictions made via tRNAScan.
(XLSX)

Table S6 Best matches of manually curated *R. solani* AG8 genes to NCBI Protein database by BLASTP.
(XLSX)

Table S7 Comparison of SignalP 4.1 predictions between *R. solani* AG8 and 86 fungal species.
(XLSX)

Table S8 Carbohydrate-active enzymes (CAZymes) present in the *R. solani* AG8 genome. (A) Annotations made via the CAZymes Analysis Toolkit. (B) Manual curation of data presented in (A). (C) Summary counts of CAZyme content of *R. solani* AG8.
(XLSX)

Table S9 Pfams comparisons between *R. solani* AG8 and species of the JGI Integrated Microbial Genomes database. Data is summarised for Pfams abundant in AG8 in (A) and presented in full in (B).
(XLS)

Table S10 Comparison of Pfam annotations between *R. solani* AG8 and AG1-IA. (A) Counts of genes with Pfam annotations for both AGs. (B) Pfams significantly enriched in AG1-IA relative to AG8. (C) Pfams significantly enriched in AG8 relative to AG1-IA. (D) Full Pfam annotations for AG1-IA. (E) Full Pfam annotations for AG8.
(XLSX)

Table S11 Summary of orthology relationships between *R. solani* AG8 and 197 species. Compared species include fungal, oomycete, insect and prokaryotic species exhibiting a wide range of pathogenicity phenotypes.
(XLSX)

Table S12 Predicted ‘effector-like’ plant pathogenicity genes of *R. solani* AG8 and data supporting their prediction.
(XLSX)

Table S13 Summary of matches in *R. solani* AG8 proteins to known plant pathogenicity motifs.
(XLSX)

Table S14 Summary of relative mRNA expression of selected predicted ‘effector-like’ genes. Selected genes were isolated from 7 dpi infected wheat versus 7 day old *in vitro* culture. (A) Raw CT values for all genes tested. (B) Genes with significantly up-regulated mRNA expression *in planta*.
(XLSX)

Table S15 Multiple alignments of the most abundant CAZyme families in *R. solani* AG8.
(XLSX)

Text S1 Methods used to assemble a haploid representation of the multinucleate genome of *R. solani* AG8.
(DOCX)

Text S2 The mitochondrial genome of *R. solani* AG8. Confirmation of circularity and correct scaffolding across internal gap of mitochondrial Scaffold_77 by PCR and its predicted mitochondrial genes and non-coding RNA regions.
(DOCX)

Text S3 *De novo* MEME search for novel motifs among the 308 *R. solani* AG8 effector candidates.
(TXT)
