# Supplementary figures and images for: Genome Sequencing and Comparative Genomics of the Broad Host-Range Pathogen Rhizoctonia solani AG8
Source: PLoS Genet. 2014 May 8;10(5):e1004281. doi: 10.1371/journal.pgen.1004281 (PMC4014442; doi:10.1371/journal.pgen.1004281)

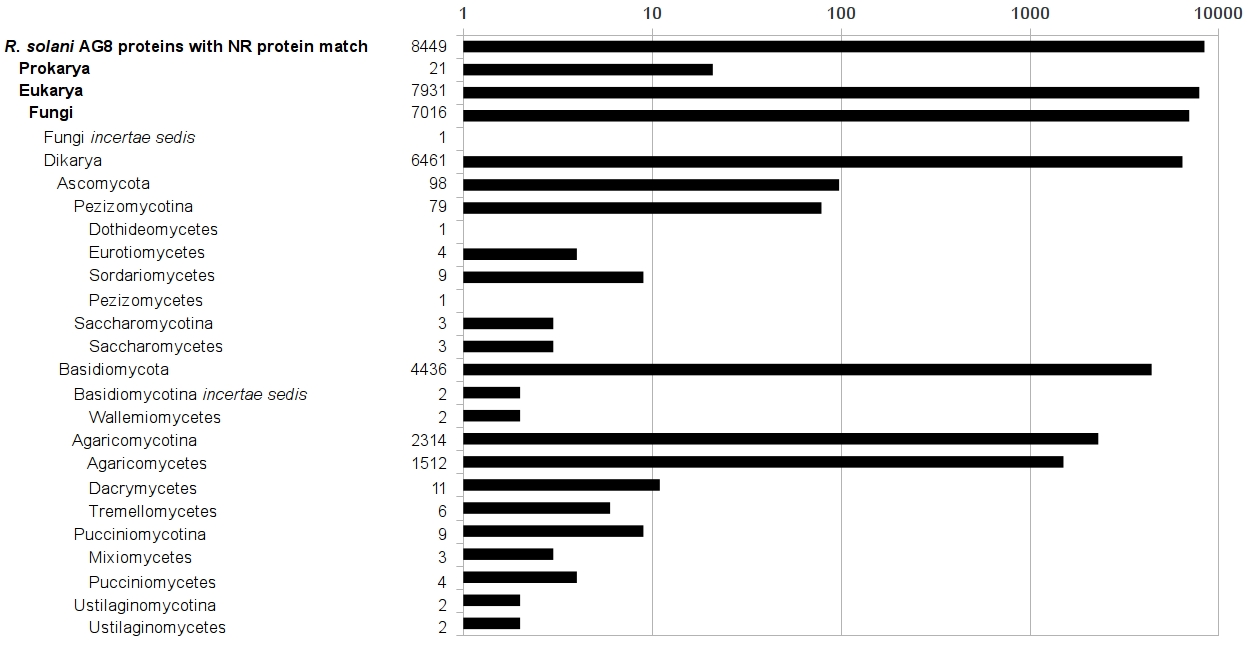

Supplement: Figure S1 — Summary of “lowest-common-ancestor” taxa assigned to 8,449 R. solani AG8 proteins by BLASTP to NCBI Protein. Higher level taxa contain protein counts both for widely-conserved R. solani AG8 proteins for which that taxon has been assigned as its lowest-common-ancestor, as well as cumulative counts for all lower-level taxa contained within. (TIF) [file pgen.1004281.s001.tif]

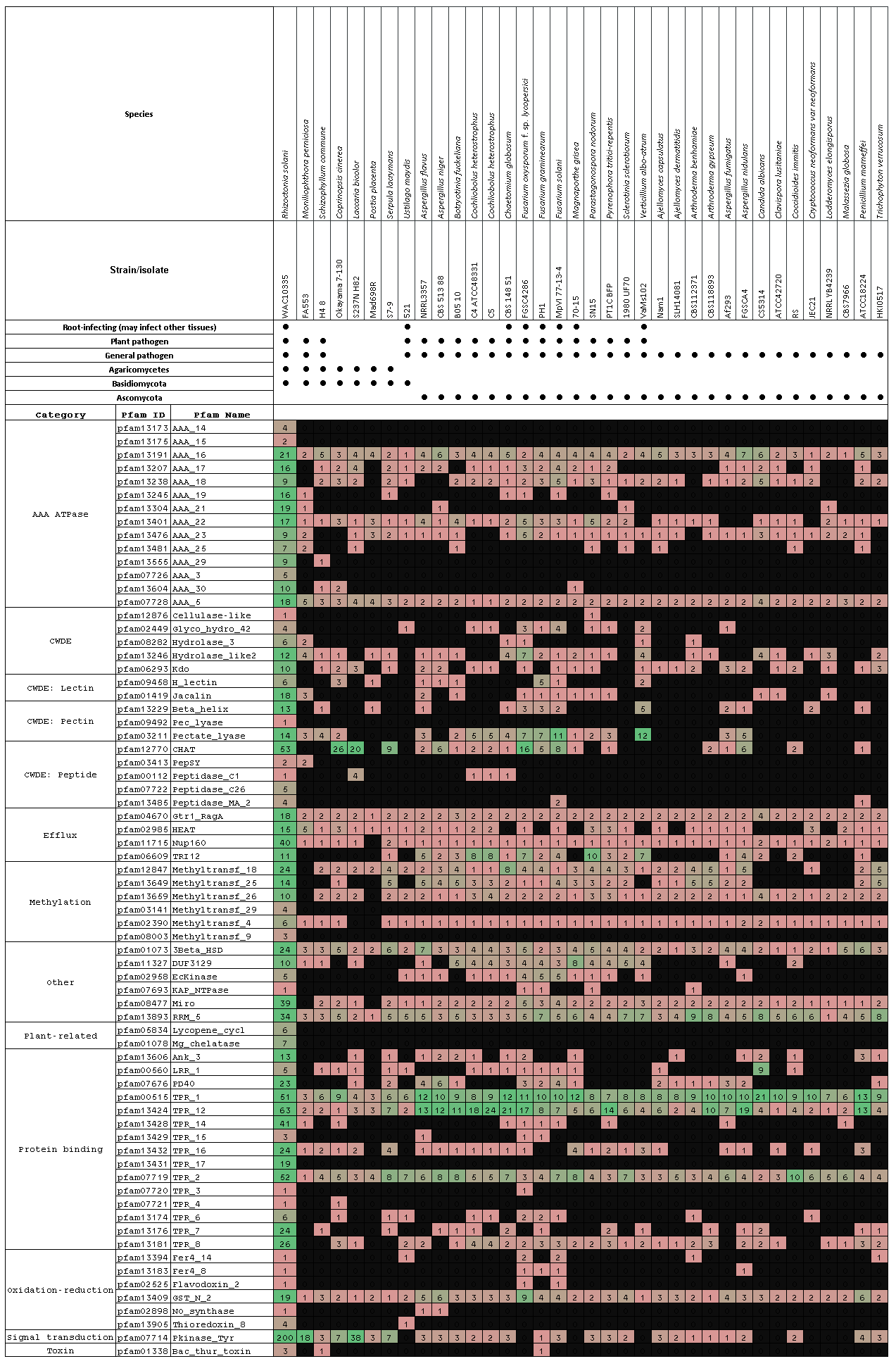

Supplement: Figure S2 — Pfams abundant in R. solani AG8 compared to species from JGI Integrated Microbial Genomes. (TIF) [file pgen.1004281.s002.tif]
