# Supplementary material for: Genome Sequencing and Comparative Genomics of the Broad Host-Range Pathogen Rhizoctonia solani AG8
Source: PLoS Genet. 2014 May 8;10(5):e1004281. doi: 10.1371/journal.pgen.1004281 (PMC4014442; doi:10.1371/journal.pgen.1004281)
Supplement: Text S1 — Methods used to assemble a haploid representation of the multinucleate genome of R. solani AG8. (DOCX) [file pgen.1004281.s018.docx]

*De novo* genome assembly using the standard paired-end, short-read libraries of current next-generation sequencing platforms were not sufficient to generating long scaffolds across complex sequence regions required to assemble the heterozygous *R. solani* AG8 genome. One way to span complex regions, whilst still operating within the read-length limitations of the low cost Illumina next-generation sequencing platform, was the use of mate-paired libraries whereby large fragments (kilobase scale) are converted to a smaller compatible fragment size via circularisation, fragmentation and purification of fragments corresponding to the original large fragment termini (Figure 1). A side effect of this process is the introduction of various contaminant read pairs (Figure 1), the most abundant and therefore most detrimental to de novo assembly are paired-end contaminants, also referred to as a “shadow library” within the mate-paired library.


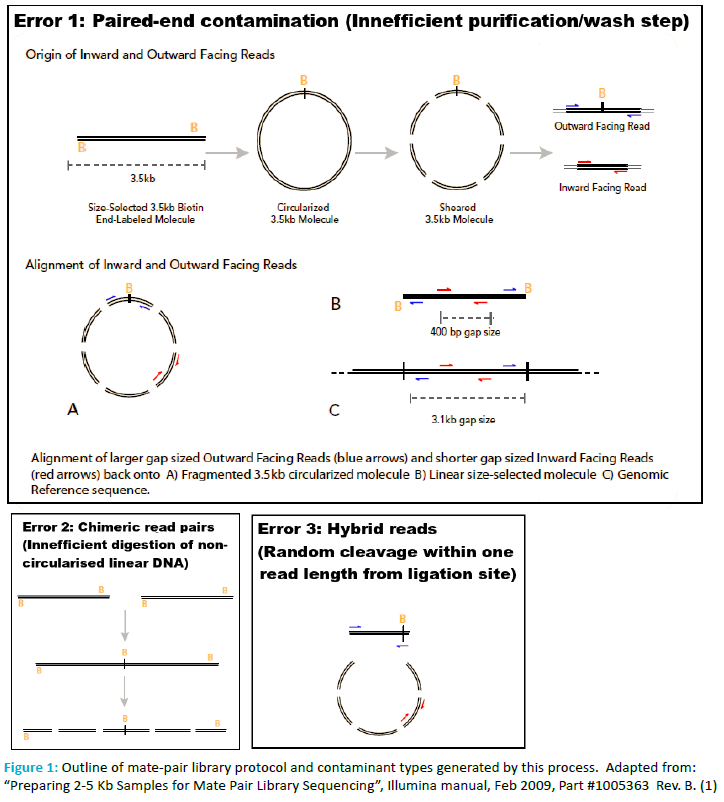


**Figure 2: The two major types of assembly errors that could potentially be introduced into a de novo assembly, either by improper use of mate-paired data (inverted duplications) or through incorrect scaffold joining/extension at heterozygous sequence regions (tandem duplications). The X and Y axis represent the same sequence compared against itself in a dot plot visualisation. The green and blue regions represent unique sequence regions flanking an error, which is indicated in red. Lines in the plot represent internal sequence matches between these regions.**

Introduction of paired-end contaminants is a major problem for accurate de novo assembly as this introduces one or more inversion errors (Figure 2). In this study we employ two methods to filter paired end or “shadow library” contamination: 1) remove reads with overlapping 3’ ends with FLASH, 2) Impose a minimum size threshold for sequences to be scaffolded/joined by mate-pairs – this must be greater than the maximum expected insert size for the shadow library (usually 1kb) (Figure 3).

**Figure 3: Simple protocol for excluding paired-end contaminants not removed by FLASH (non-overlapping 3’ ends). This figure depicts correct mate-paired reads (blue) and contaminant paired-end reads (red) aligned to pre-scaffolded sequences. Sequence lengths considered for joining into larger scaffolds on the basis of mate-paired paring relationships are required to be greater than the maximum expected “shadow-library” insert size. In most cases this threshold is around 1 kb.**

**Figure 4: Method for correction of tandem duplications caused by scaffolding errors at heterozygous regions. At each of the two polymorphic sites depicted here, there are two heterozygous alleles, the less frequent is indicated in red and the majority in green.**

Methods which fill in stretches of unknown bases (gaps) introduced between scaffolding joins, with k-mer based overlap algorithms, tend to perform poorly when the scaffolded region is heterozygous. This is because minor sequence differences cause the k-mer overlap extension not to meet in the middle and can lead to over-extension that causes localised tandem duplication with minor sequence differences. Our method for correcting these errors was to test scaffolded regions during assembly rounds for overlap with CAP3 (min 75% identity, any length overhang allowable). Then after final assembly for tandem overlap with HaploMerger. As tandem overlap errors from Illumina short reads are likely to occur roughly within 100bp, the parameters of HaploMerger were adjusted to this shorter length. Merging of heterozygous regions did not always result in the most abundant haplotypes being represented in the haploid consensus, therefore alignment of genomic short reads to scaffold sequences was performed and a majority consensus sequences was recalculated (Figure 4).

As *R. solani* contains RIP mutations (see main manuscript), the calculation of the majority consensus was not always conducive to representing the maximum coding potential of gene regions. RIP mutations often introduce stop codons and if consensus haplotypes in genes were a RIP-Stop mutation, the *in silico* translation of those gene regions would be truncated nonsense products. It would be possible for RIP-truncated gene products of a particular gene to be the most frequently found alleles on most nuclei, but for a small number of nuclei to have escaped RIP and produce full length gene products. Therefore to represent the full coding potential of all gene regions we have applied additional methods to adjust the majority consensus sequences at RIP polymorphic sites to a pre-RIP state (Figure 5).

**Figure 5: RIP acts upon DNA heteroduplexes, randomly converting cytosines to thiamines, indicated in the upper part of this diagram in red. In the lower part of this diagram, the random introduction of thiamines leads to the accumulation of TGA, TAG and TAA nonsense mutations (red). If the majority consensus in a protein-coding gene region contains RIP-Stop genes in some but not all nuclei, then there is still potential for full length gene products to be produced. In cases where genomic short reads indicate that pre-RIP alleles (green) exist but not in the majority, the consensus sequence was reverted to its preRIP state instead of the most frequent allele.**

The correction of the above assembly errors, as well as annotation of genes in a way that corrects for the propensity of fungal UTR overlaps and mistakes in automated gene prediction, is essential to the accurate functional analysis of a whole genome. Whole genome functional analysis relies on the detection and accurate quantitation of amino-acid domains with associated functional ontologies (i.e. GO/PFAM/etc). The types of gene annotation errors are outlined in Figure 6 and the ways in which this can impact upon domain detection and quantitation is outlined in Figure 7 below.

**Figure 6: Types of gene annotation errors. Bars indicate coding exon (CDS) annotations.**

**Figure 7: Impact of gene annotation errors on the detection and/or quantitation of functionally associated domains (indicated in red).**
