# Supplementary material for: Genome Sequencing and Comparative Genomics of the Broad Host-Range Pathogen Rhizoctonia solani AG8
Source: PLoS Genet. 2014 May 8;10(5):e1004281. doi: 10.1371/journal.pgen.1004281 (PMC4014442; doi:10.1371/journal.pgen.1004281)
Supplement: Text S2 — The mitochondrial genome of R. solani AG8. Confirmation of circularity and correct scaffolding across internal gap of mitochondrial Scaffold_77 by PCR and its predicted mitochondrial genes and non-coding RNA regions. (DOCX) [file pgen.1004281.s019.docx]

# Confirmation of circularity by PCR

## Primer pairs

**5prime_flank_R**

GACCGATCGCAATTCTAAGC

Length: 20 bp

Tm: 59.8 °C

GC: 50.0 %

ANY: 6.0

SELF: 2.0

3' Stability: 7.5 ΔG

**3prime_flank_F**

CTTCCAAGGAGTGGAAGCTC

Length: 20 bp

Tm: 59.0 °C

GC: 55.0 %

ANY: 7.0

SELF: 2.0

3' Stability: 7.9 ΔG

**internal5primeflankF**

CCTTCGGCTGGTAATCAGAA

Length: 20 bp

Tm: 60.2 °C

GC: 50.0 %

ANY: 5.0

SELF: 2.0

3' Stability: 7.0 ΔG

**internal3primeflankR**

GCCGGCGGTATTTTAAGACT

Length: 20 bp

Tm: 60.5 °C

GC: 50.0 %

ANY: 6.0

SELF: 3.0

3' Stability: 6.1 ΔG

## In silico PCR

### Primer pair combinations tested

| **Primer combination** | **Fwd** | **Rev** | **Amplicon test range (bp)** |
| --- | --- | --- | --- |
| maingap | GACCGATCGCAATTCTAAGC | CTTCCAAGGAGTGGAAGCTC | 0-10000000 |
| internalgap | CCTTCGGCTGGTAATCAGAA | GCCGGCGGTATTTTAAGACT | 0-10000000 |
| 3Flank_Int5 | CTTCCAAGGAGTGGAAGCTC | CCTTCGGCTGGTAATCAGAA | 0-10000000 |
| 3Flank_Int3 | CTTCCAAGGAGTGGAAGCTC | GCCGGCGGTATTTTAAGACT | 0-10000000 |
| 5Flank_Int5 | GACCGATCGCAATTCTAAGC | CCTTCGGCTGGTAATCAGAA | 0-10000000 |
| 5Flank_Int3 | GACCGATCGCAATTCTAAGC | GCCGGCGGTATTTTAAGACT | 0-10000000 |
| 5Flank | GACCGATCGCAATTCTAAGC | GACCGATCGCAATTCTAAGC | 0-10000000 |
| 3Flank | CTTCCAAGGAGTGGAAGCTC | CTTCCAAGGAGTGGAAGCTC | 0-10000000 |
| Int5 | CCTTCGGCTGGTAATCAGAA | CCTTCGGCTGGTAATCAGAA | 0-10000000 |
| Int3 | GCCGGCGGTATTTTAAGACT | GCCGGCGGTATTTTAAGACT | 0-10000000 |

### *In silico* amplicons

| Scaffold_77 | maingap | + | 48613 | 107122 | 58510/0-10000000 |
| --- | --- | --- | --- | --- | --- |
| Scaffold_77 | maingap | + | 27475 | 107122 | 79648/0-10000000 |
| Scaffold_77 | maingap | + | 27475 | 39816 | 12342/0-10000000 |
| Scaffold_77 | maingap | + | 27475 | 37495 | 10021/0-10000000 |
| Scaffold_77 | internalgap | + | 134387 | 138734 | 4348/0-10000000 |
| Scaffold_77 | internalgap | + | 133797 | 138734 | 4938/0-10000000 |
| Scaffold_77 | internalgap | + | 130793 | 138734 | 7942/0-10000000 |
| Scaffold_77 | internalgap | + | 45926 | 138734 | 92809/0-10000000 |
| Scaffold_77 | 3Flank_Int5 | - | 45926 | 107122 | 61197/0-10000000 |
| Scaffold_77 | 5Flank_Int5 | + | 48613 | 58181 | 9569/0-10000000 |
| Scaffold_77 | 5Flank_Int5 | + | 27475 | 58181 | 30707/0-10000000 |
| Scaffold_77 | 5Flank_Int5 | + | 27475 | 27695 | 221/0-10000000 |
| Scaffold_77 | 5Flank_Int5 | - | 45926 | 82289 | 36364/0-10000000 |
| Scaffold_77 | 5Flank_Int5 | - | 45926 | 59122 | 13197/0-10000000 |
| Scaffold_77 | 5Flank_Int3 | + | 48613 | 138734 | 90122/0-10000000 |
| Scaffold_77 | 5Flank_Int3 | + | 27475 | 138734 | 111260/0-10000000 |
| Scaffold_77 | 5Flank | + | 48613 | 82289 | 33677/0-10000000 |
| Scaffold_77 | 5Flank | + | 48613 | 59122 | 10510/0-10000000 |
| Scaffold_77 | 5Flank | + | 27475 | 82289 | 54815/0-10000000 |
| Scaffold_77 | 5Flank | + | 27475 | 59122 | 31648/0-10000000 |
| Scaffold_77 | 5Flank | - | 48613 | 82289 | 33677/0-10000000 |
| Scaffold_77 | 5Flank | - | 48613 | 59122 | 10510/0-10000000 |
| Scaffold_77 | 5Flank | - | 27475 | 82289 | 54815/0-10000000 |
| Scaffold_77 | 5Flank | - | 27475 | 59122 | 31648/0-10000000 |
| Scaffold_77 | Int5 | + | 45926 | 58181 | 12256/0-10000000 |
| Scaffold_77 | Int5 | - | 45926 | 58181 | 12256/0-10000000 |

## PCR results


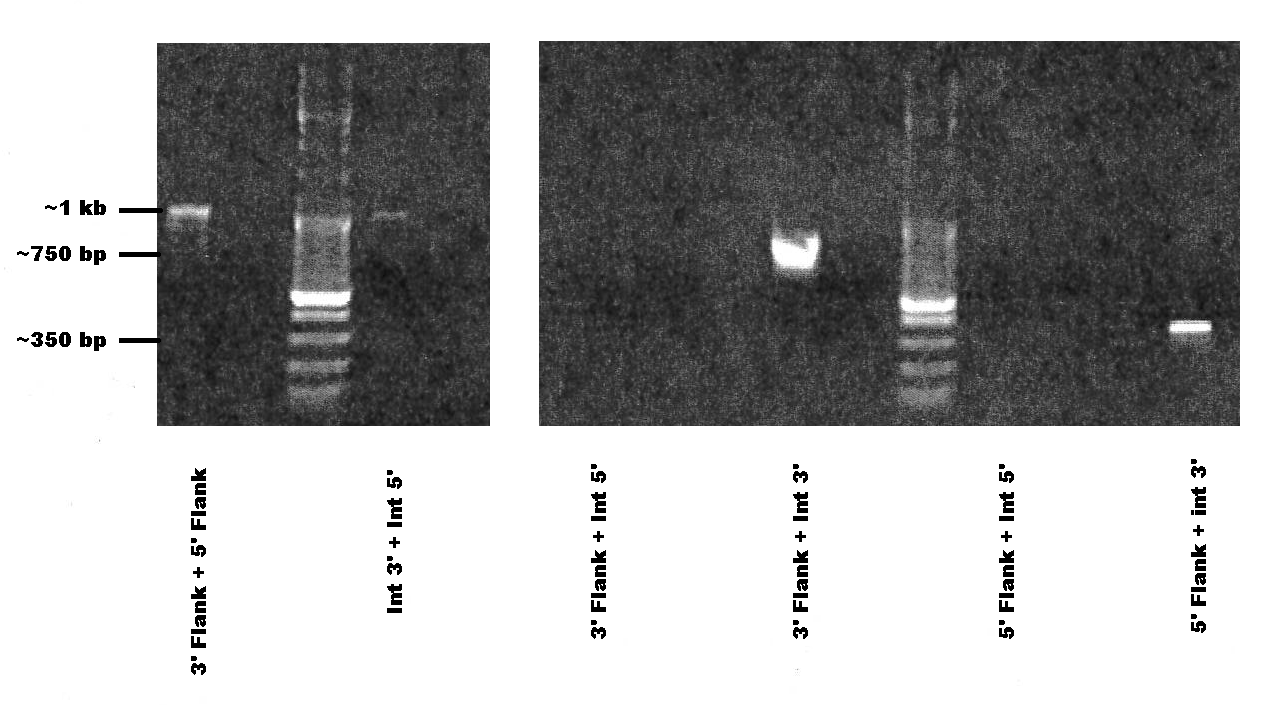


# Mitochondrial genome annotation


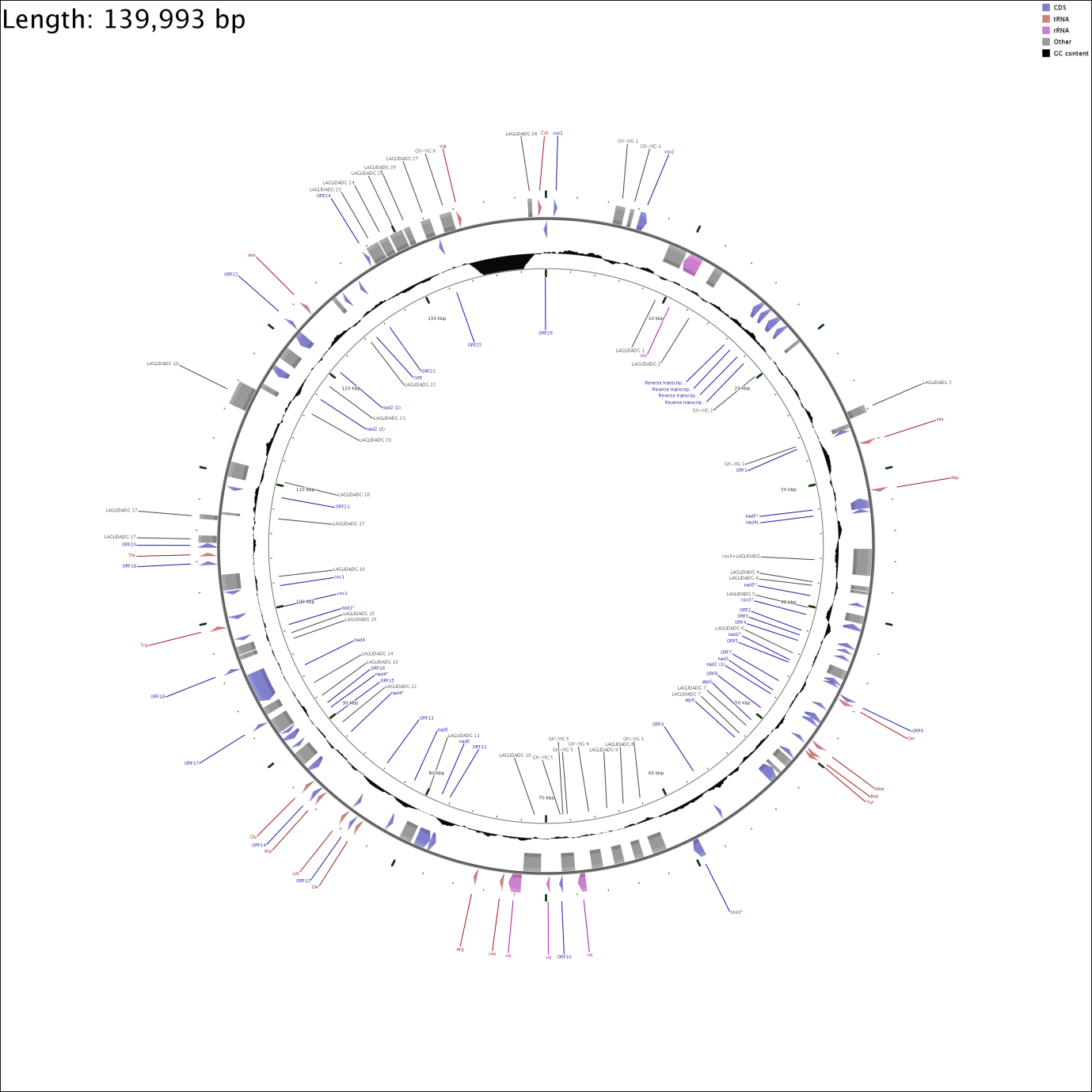


## Genes, Endonuclease repeats, tRNAs, rRNAs

| seqname | feature | start | end | strand |
| --- | --- | --- | --- | --- |
| cox2 | CDS | 522 | 753 | + |
| GIY-YIG 1 | other | 4526 | 5155 | + |
| GIY-YIG 1 | other | 5482 | 5748 | + |
| cox2 | CDS | 6170 | 6747 | + |
| LAGLIDADG 1 | other | 8667 | 9965 | - |
| rns | rRNA | 10025 | 11200 | - |
| LAGLIDADG 2 | other | 12170 | 12802 | - |
| Reverse transcriptase | CDS | 15915 | 16388 | - |
| Reverse transcriptase | CDS | 16581 | 17000 | - |
| Reverse transcriptase | CDS | 17358 | 17906 | - |
| Reverse transcriptase | CDS | 18197 | 18589 | - |
| GIY-YIG 2 | other | 19705 | 19932 | - |
| LAGLIDADG 3 | other | 25671 | 26186 | + |
| GIY-YIG 2 | other | 26423 | 26731 | - |
| ORF1 | CDS | 26724 | 26897 | - |
| nad5* | CDS | 31641 | 32384 | - |
| nad4L | CDS | 32384 | 32650 | - |
| cox3+LAGLIDADG | other | 35200 | 37062 | - |
| LAGLIDADG 4 | other | 37821 | 38123 | - |
| LAGLIDADG 4 | other | 38165 | 38368 | - |
| nad5* | CDS | 39068 | 39202 | - |
| LAGLIDADG 5 | other | 39844 | 40407 | - |
| cox3* | CDS | 40537 | 40929 | - |
| ORF2 | CDS | 41944 | 42243 | - |
| ORF3 | CDS | 42386 | 42637 | - |
| ORF4 | CDS | 42886 | 43095 | - |
| LAGLIDADG 6 | other | 43789 | 44424 | - |
| nad2* | CDS | 44577 | 44885 | - |
| ORF5 | CDS | 44899 | 45003 | - |
| ORF6 | CDS | 45406 | 45663 | + |
| ORF7 | CDS | 46576 | 46791 | - |
| nad3 | CDS | 47392 | 47784 | - |
| nad2 (1) | CDS | 47787 | 48107 | - |
| ORF8 | CDS | 49259 | 49480 | - |
| atp6 | CDS | 50501 | 50809 | - |
| LAGLIDADG 7 | other | 50984 | 51559 | - |
| LAGLIDADG 7 | other | 52026 | 52244 | - |
| atp6 | CDS | 52252 | 53025 | - |
| ORF9 | CDS | 57033 | 57119 | - |
| cox1* | CDS | 59328 | 59915 | + |
| GIY-YIG 3 | other | 61540 | 62535 | - |
| LAGLIDADG 8 | other | 63191 | 63823 | - |
| LAGLIDADG 9 | other | 64559 | 65260 | - |
| GIY-YIG 4 | other | 66054 | 66836 | - |
| rnl | rRNA | 67350 | 67907 | + |
| GIY-YIG 5 | other | 67988 | 68458 | - |
| GIY-YIG 5 | other | 68455 | 68730 | - |
| GIY-YIG 5 | other | 68720 | 68920 | - |
| ORF10 | CDS | 68927 | 69091 | + |
| rnl | rRNA | 69771 | 69961 | + |
| LAGLIDADG 10 | other | 70346 | 71569 | - |
| rnl | rRNA | 71635 | 72480 | + |
| ORF11 | CDS | 77914 | 78333 | - |
| nad5 | CDS | 78338 | 79312 | - |
| LAGLIDADG 11 | other | 79545 | 80399 | - |
| nad5 | CDS | 81237 | 81530 | - |
| ORF12 | CDS | 83600 | 83731 | + |
| ORF13 | CDS | 84008 | 84121 | - |
| ORF14 | CDS | 86611 | 86901 | + |
| nad4* | CDS | 87816 | 88271 | - |
| LAGLIDADG 12 | other | 88607 | 89605 | - |
| ORF15 | CDS | 89847 | 90044 | - |
| nad4* | CDS | 90452 | 90895 | - |
| ORF16 | CDS | 91026 | 91253 | - |
| LAGLIDADG 13 | other | 91363 | 92439 | - |
| ORF17 | CDS | 92440 | 92592 | + |
| LAGLIDADG 14 | other | 92863 | 93417 | - |
| nad4 | CDS | 93678 | 95909 | - |
| ORF18 | CDS | 96456 | 96692 | + |
| LAGLIDADG 15 | other | 97032 | 97331 | - |
| LAGLIDADG 15 | other | 97396 | 97968 | - |
| nad1* | CDS | 98309 | 98476 | - |
| cox1 | CDS | 99808 | 100098 | - |
| cox1 | CDS | 101600 | 101803 | - |
| LAGLIDADG 16 | other | 101924 | 103024 | - |
| ORF19 | CDS | 103741 | 103998 | + |
| ORF20 | CDS | 104881 | 105186 | + |
| LAGLIDADG 17 | other | 105207 | 105683 | + |
| LAGLIDADG 17 | other | 106742 | 107050 | + |
| LAGLIDADG 17 | other | 107161 | 107328 | - |
| ORF21 | CDS | 108956 | 109078 | - |
| LAGLIDADG 18 | other | 109784 | 110848 | - |
| LAGLIDADG 19 | other | 114407 | 115999 | + |
| LAGLIDADG 20 | other | 116260 | 116610 | - |
| nad2 (2) | CDS | 117548 | 118111 | - |
| LAGLIDADG 21 | other | 118699 | 119580 | - |
| nad2 (2) | CDS | 120256 | 120975 | - |
| ORF22 | CDS | 120974 | 121135 | + |
| LAGLIDADG 22 | other | 124049 | 124351 | - |
| cytb | CDS | 124681 | 124938 | - |
| ORF23 | CDS | 126111 | 126236 | - |
| ORF24 | CDS | 127548 | 127769 | + |
| LAGLIDADG 23 | other | 127888 | 128739 | + |
| LAGLIDADG 24 | other | 128816 | 129433 | + |
| LAGLIDADG 25 | other | 129592 | 130461 | + |
| LAGLIDADG 26 | other | 130580 | 130987 | + |
| LAGLIDADG 27 | other | 131746 | 132384 | + |
| ORF25 | CDS | 132381 | 132524 | - |
| GIY-YIG 6 | other | 133031 | 133810 | + |
| LAGLIDADG 28 | other | 138807 | 139079 | + |
| ORF26 | CDS | 139892 | 139993 | - |
| Leu | tRNA | 72891 | 72973 | + |
| Arg | tRNA | 74663 | 74734 | + |
| Gln | tRNA | 83143 | 83219 | + |
| Lys | tRNA | 84293 | 84365 | + |
| Arg | tRNA | 86285 | 86357 | + |
| Gly | tRNA | 87412 | 87483 | + |
| Trp | tRNA | 99495 | 99565 | + |
| Thr | tRNA | 104401 | 104473 | + |
| Asn | tRNA | 122429 | 122500 | + |
| Val | tRNA | 134219 | 134289 | + |
| Cys | tRNA | 139558 | 139630 | + |
| Tyr | tRNA | 50006 | 49923 | + |
| Met | tRNA | 49783 | 49710 | + |
| Met | tRNA | 49197 | 49125 | + |
| Ser | tRNA | 45878 | 45795 | + |
| Asp | tRNA | 31325 | 31253 | + |
| His | tRNA | 28083 | 28012 | + |
